# Single-cell profiling of the human endometrium in polycystic ovary syndrome

**DOI:** 10.1038/s41591-025-03592-z

**Published:** 2025-03-20

**Authors:** Gustaw Eriksson, Congru Li, Tina Gorsek Sparovec, Anja Dekanski, Sara Torstensson, Sanjiv Risal, Claes Ohlsson, Angelica Lindén Hirschberg, Sophie Petropoulos, Qiaolin Deng, Elisabet Stener-Victorin

**Affiliations:** 1 Department of Physiology and Pharmacology, Karolinska Institutet, Stockholm, Sweden; 2 Department of Clinical Science, Intervention and Technology, Division of Obstetrics/Gynecology, Karolinska Institutet, Stockholm, Sweden; 3 Department of Drug Treatment, Sahlgrenska University Hospital, Region Västra Götaland, Gothenburg, Sweden; 4 Department of Internal Medicine and Clinical Nutrition, Institute of Medicine, Sahlgrenska Osteoporosis Centre, Centre for Bone and Arthritis Research at the Sahlgrenska Academy, University of Gothenburg, Gothenburg, Sweden; 5 Department of Women’s and Children’s Health, Karolinska Institutet, Stockholm, Sweden; 6 Department of Gynecology and Reproductive Medicine, Karolinska University Hospital, Stockholm, Sweden; 7 Faculty of Medicine, Département de Médecine, Université de Montréal, Montréal, Québec Canada; 8 Centre de Recherche du Centre Hospitalier de l’Université de Montréal, Axe Immunopathologie, Montréal, Québec Canada; 9 Department of Molecular Biosciences, Wenner-Gren Institute, Stockholm University, Stockholm, Sweden

**Keywords:** Endocrine reproductive disorders, Gene expression, Endocrine reproductive disorders

## Abstract

Polycystic ovary syndrome (PCOS) has a negative effect on the receptivity of the endometrium to embryo implantation and increases the risk of miscarriage and endometrial cancer. The cellular and molecular heterogeneity of the endometrium in women with PCOS has not been well studied. Our study presents a comprehensive cellular atlas of the endometrium during the proliferative phase in women with PCOS characterized by overweight and obesity, hyperandrogenism and insulin resistance compared with controls of similar age, weight and body mass index. Analysis of 247,791 isolated endometrial nuclei from 27 biopsies (5 controls and 12 PCOS cases at baseline and 7 after 16 weeks of metformin and 3 after lifestyle intervention) revealed cell-type-specific disease signatures and variations in cellular composition and localization. Samples taken after 16 weeks of metformin treatment and lifestyle management showed extensive recovery of disease-specific endometrial signatures. We linked the specific role of each cell type to clinical features such as hyperandrogenism and insulin resistance, and specific cell types to risk of endometrial and metabolic disease. In addition, potential therapeutic targets such as integrin inhibitors were identified and the role of metformin in restoring endometrial health in patients with PCOS was highlighted. Our findings lay the groundwork to significantly advance the understanding of PCOS-specific endometrial dysfunction for future targeted therapies.

## Abstract (web summary)

A transcriptomic atlas of the endometrium of women with polycystic ovary syndrome uncovers both disease signatures and potential therapeutic targets.

## Main

Polycystic ovary syndrome (PCOS) affects 11–13% of reproductive-aged women and caused an economic burden of US$8 billion in 2020 (refs. ^1,2^). PCOS is a leading cause of anovulatory infertility and is linked to comorbidities such as insulin resistance, type 2 diabetes (T2D) and endometrial cancer^3^. Key reproductive features include hyperandrogenism, anovulation and menstrual irregularities, which are exacerbated by obesity^3^. Women with PCOS experience implantation failure, early and mid-pregnancy miscarriages and higher risks of gestational diabetes, hypertension, pre-eclampsia and intrauterine growth restriction compared with women without PCOS^4^—conditions associated with endometrial dysfunction.

The PCOS-endometrium exhibits unopposed estrogen stimulation and progesterone resistance^5,6^, impairing decidualization and potentially altering immune cell function^7–9^. These changes, alongside altered gene expression, are associated with reduced endometrial receptivity, early miscarriages, poor pregnancy outcomes and increased risk of endometrial cancer. An altered immune environment, compared with women without PCOS, is also expected^10^. Although these findings suggest intrinsic endometrial dysfunction and a distinct PCOS phenotype, little is known about how specific cell types—such as epithelial, stromal, endothelial, glandular, smooth muscle and immune cells—contribute to this dysfunction or whether specific cell types play a dominant role.

Current PCOS treatments target symptoms without addressing underlying mechanisms. Lifestyle management, the first-line treatment for overweight or obese women with PCOS^1,3^, improves insulin signaling and modulates androgen, estrogen-α and progesterone receptor expression^6,9,11^. Metformin, used when lifestyle changes are insufficient^1,3^, alleviates insulin resistance and hyperandrogenism, reversing androgen effects on insulin receptor-I and glucose-transporter type 4 (GLUT4) while improving immune alterations^4,12^. However, its impact on cell-type-specific endometrial dysfunction in PCOS remains unclear.

In the present study, we applied single-nuclei RNA sequencing (snRNA-seq) and spatial transcriptomics to create a cellular and transcriptional atlas of the proliferative-phase endometrium in overweight and obese, insulin-resistant women with and without PCOS. This approach identified cell-type-specific molecular disease signatures, variations in cellular composition and the spatial localization of PCOS-specific cells. Remarkably, some PCOS-specific endometrial signatures were partially restored after 16 weeks of metformin and lifestyle management.

## Results

### Clinical characteristics

**Fig. 1:**
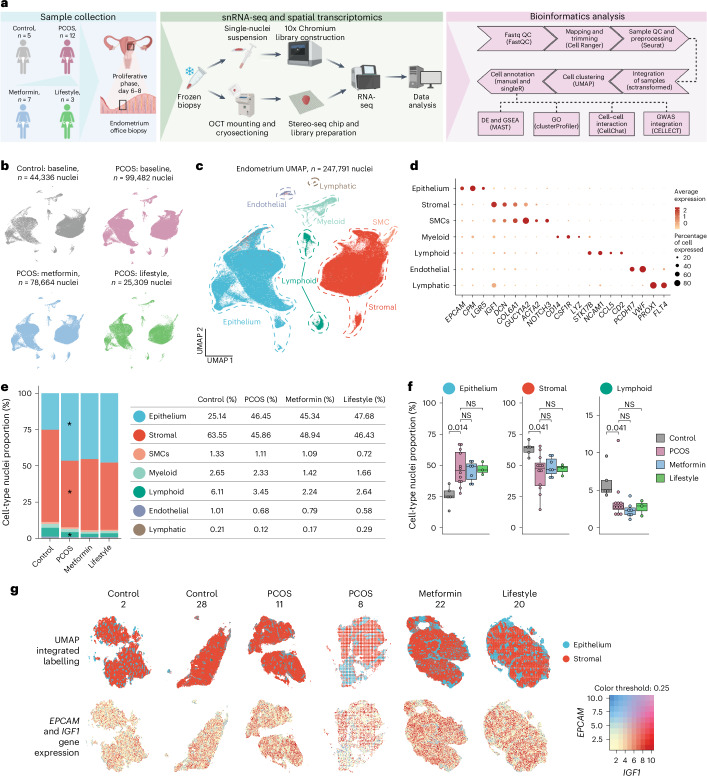
Endometrial single-cell profiling in women with PCOS and controls. **a**, Schematic illustration of the included participants, endometrial biopsies collected in proliferative phase days 6–8, snRNA-seq with 10x Genomics and spatial transcriptomics with Stereo-seq and bioinformatic analysis pipeline. GSEA, gene set enrichment analyses; QC, quality control. **b**, Color code and number of captured nuclei for snRNA-seq of control and PCOS-endometrium at baseline and after 16 weeks of treatment with metformin or lifestyle intervention. **c**, UMAP projections of the integrated snRNA-seq data from 27 participants (*n* = 247,791 nuclei) showing 7 cell-type-specific clusters. **d**, Dotplots showing the log(transformed) gene expression of cell-type markers characterizing each main cluster. **e**, Bar plot and table showing the proportion of major cell clusters from endometrial biopsies of the proliferative phase at baseline (control, *n* = 5; PCOS, *n* = 12; and after 16 weeks of intervention: PCOS-metformin, *n* = 7; PCOS-lifestyle, *n* = 3; *BH-adjusted *P* < 0.05 following a two-sided Mann–Whitney *U*-test). **f**, Boxplot showing the differences in the nuclei proportion (percentage of cell nuclei) of epithelial, stromal and lymphoid cells between women with and women without PCOS at baseline (BH-adjusted exact *P* value after two-sided Mann–Whitney *U*-test) and the changes from baseline to after 16 weeks of metformin or lifestyle, respectively, in women with PCOS (BH-adjusted *P* exact value after two-sided Wilcoxon’s signed-rank test). NS, not significant. The line inside the box indicates the median value and the box delimits the 25th and 75th percentiles. The smallest and largest values are indicated by the lines outside the box. **g**, Spatial transcriptomic with Stereo-seq of integrated labeling and the main markers for epithelial and stromal main clusters from two control individuals, two with PCOS, one with PCOS after 16 weeks of metformin and one with PCOS after lifestyle management. Schematic in **a** created using BioRender.com.

Superficial endometrial biopsies from the functionalis layer were collected during the proliferative phase (days 6–8) from 12 women with PCOS (all but one meeting the Rotterdam criteria) and 5 controls of a similar age, weight and body mass index (BMI) (Fig. 1a and Supplementary Table 1a,b). After baseline assessment, ten participants with PCOS underwent a 16-week randomized trial (metformin: *n* = 7; lifestyle: *n* = 3) (Fig. 1a). Participants were medication free for ≥3 months, except five PCOS cases using Provera for bleeding induction (Supplementary Table 1b). PCOS participants exhibited hyperandrogenism (elevated Ferrima–Gallwey scores, dehydroepiandrosterone (DHEA) and androstenedione) and insulin resistance (higher homeostatic model assessment for insulin resistance (HOMA-IR)). Endometrial thickness and progesterone levels confirmed proliferative-phase sampling (Supplementary Table 1a,b). Metformin reduced antral follicle count, androstenedione, testosterone and a free androgen index, whereas lifestyle intervention showed no significant hormonal changes. Menstrual frequency improved in one of three lifestyle and three of seven metformin participants (Supplementary Table 1b), with shifts to regular cycles or shorter intervals.

### A single-nucleus atlas of the human PCOS-endometrium

We generated a single-nuclei map of human endometrial biopsies from all participants, identifying cell-type-specific disease signatures, compositional variations and transcriptomic changes after 16 weeks of metformin or lifestyle intervention. Single nuclei extracted from 27 biopsies were processed for snRNA-seq using the 10x Genomics protocol (Fig. 1a). Spatial transcriptomics on six samples (two controls, two PCOS-baseline, one PCOS-metformin, one PCOS-lifestyle) validated localization and transcriptomic changes after treatment (Fig. 1a).

**Extended Data Fig. 1:**
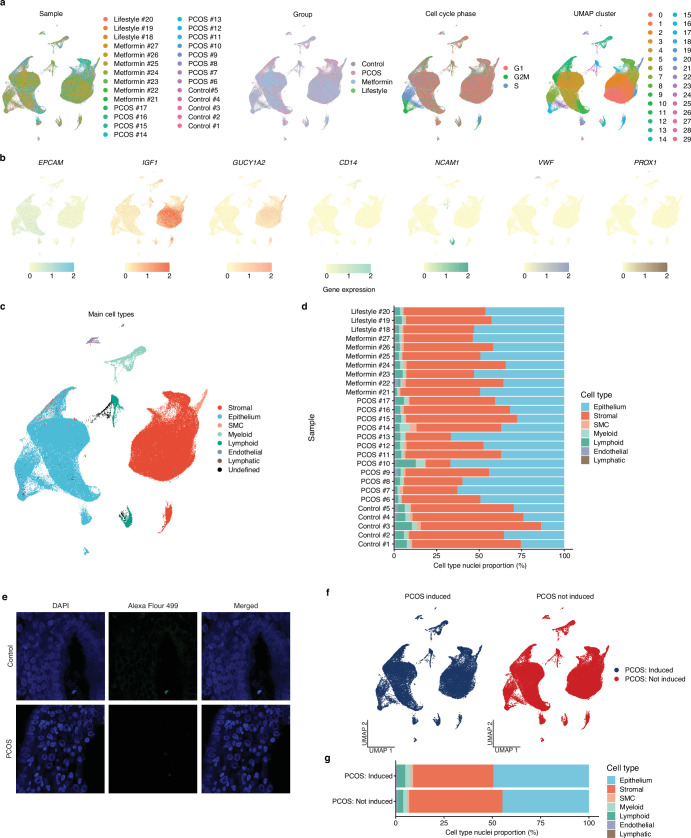
snRNA-seq individual samples and quality control. **a)** Uniform Manifold Approximation and Projections (UMAP) of 27 integrated samples, showing sample ID’s, grouping, cell cycle phase and UMAP clusters. **b)** UMAP projections of integrated samples and gene expression of the major cell type markers *EPCAM* for epithelium, *IGF1* for stroma, *GUCY1A2* for uterine smooth muscle cells, *CD14* for myeloid cells, *NCAM1* for lymphoid celss, *VWF* for endothelium and *PROX1* for lymphatic cells. **c)** UMAP projection of major cell types including undefined nuclei. **d)** Bar plot of the nuclei proportions of the main cell types in the 27 samples. **e)** Fluorescence microscopy control images of one PCOS and one control endometrial tissue for estrogen receptor alpha (Fig. 2f) using Rabbit IgG Isotype Control antibody. **f**) Uniform Manifold Approximation and Projection (UMAP) projections of integrated snRNA-seq data from 5 women with PCOS and Provera-induced bleeding and 7 women with PCOS with non-induced bleeding showing seven cell type-specific clusters. **g**) Bar plot of the nuclei proportion of major cell types of 5 samples with Provera-induced bleeding and 7 with non-induced bleeding.

After quality control, 247,791 nuclei were analyzed: 44,336 from controls, 99,482 from PCOS-baseline, 78,664 from PCOS-metformin and 25,309 from PCOS-lifestyle (Fig. 1b,c and Supplementary Table 1c–e). Seven main cell clusters were identified based on known markers^13–15^: stromal cells (*n* = 124,055), smooth muscle cells (SMCs; *n* = 2,737), epithelial cells (*n* = 105,095), lymphoid and myeloid immune cells (*n* = 13,596), endothelial cells (*n* = 2,308) and lymphatic cells (*n* = 2,308) (Fig. 1c,d and Extended Data Fig. 1a–d). Importantly, no differences in reads per nucleus, nuclei count or nuclei proportions were observed between induced and noninduced bleeding samples (Supplementary Table 1 and Extended Data Fig. 1f,g). Although endometrial biopsies were taken at the same phase of the menstrual cycle in all participants, PCOS biopsies had more epithelial cells and fewer stromal and lymphoid cells compared with controls, with no significant changes post-intervention (Fig. 1e,f). The cell-type proportions were compared with proliferative endometrium from control and endometriosis biopsies in the Human Endometrial Cell Atlas^16^, revealing similar patterns (Supplementary Table 1g).

**Extended Data Fig. 2:**
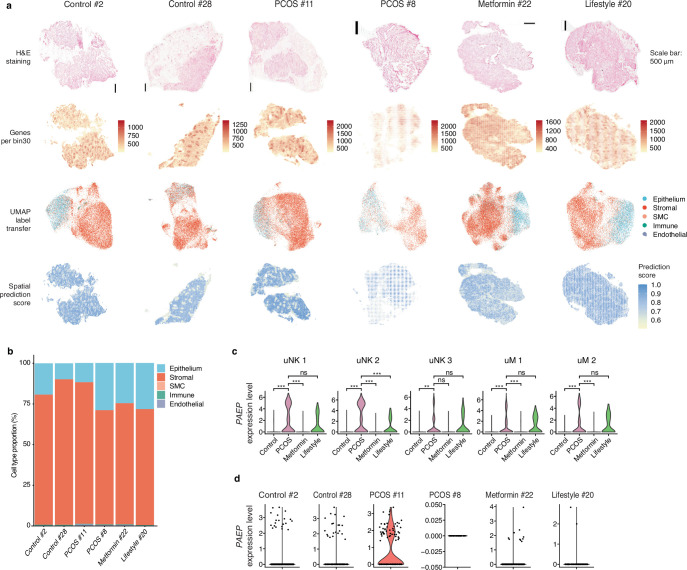
Spatial transcriptomic quality controls. **a)** From the top, spatial maps of haematoxylin and eosin (H&E) staining of adjacent sections, genes per bin30, Uniform manifold approximation and projections (UMAP) of UMAP label transfer-based cell type labelling and prediction scores of the cell type labelling of six samples: 2 controls, 2 PCOS, 1 PCOS-metformin and 2 PCOS-lifestyle. **b)** Bar plot of cell type proportions of bin30 after labelling by UMAP label transfer. **c)** Violin plots of the immunolabeled spatial bin30 of *PAEP* expression per sample and of log-transformed *PAEP* expression in the single nuclei immune cluster in the control, PCOS, PCOS-metformin and PCOS-lifestyle groups in uNK 1–3 and uM 1-2. Statistics: DEG analysis by two-sided MAST between log-transformed gene expression between groups. Benjamini-Hochberg (BH) adjusted *P* < 0.05 and a log_2_-foldchange >0.5 is considered significant. ***, BH adjusted *P* < 0.001 in DEG analysis. **d)** Violin plots of immunolabeled spatial bin30 of *PAEP* expression per sample. Source data

Spatial transcriptomics confirmed cell-type locations, validated marker genes used for cluster annotation and revealed spatial location of cellular subpopulations (Supplementary Table 1h), highlighting distinct clusters of epithelial (*EPCAM*) and stromal cells (*IGF1*) in samples from two controls, two PCOS and one PCOS-metformin and one PCOS-lifestyle (Fig. 1g and Extended Data Fig. 2a,b). Immune and endothelial cells were detected but were less prominent as a result of their lower abundance. These findings provide a detailed transcriptional and spatial map of endometrial alterations in PCOS.

### Spatiotemporal alterations in the proliferative epithelium

**Fig. 2:**
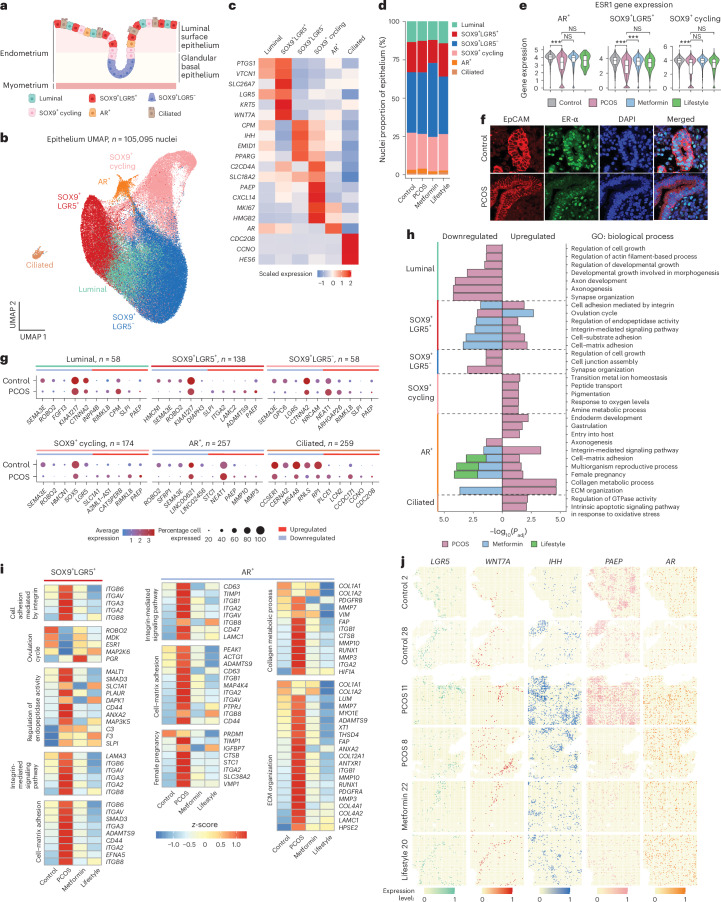
Temporal and spatial characterization of endometrial epithelial cells and effect of metformin and lifestyle in women with PCOS. **a**, Schematic illustration of epithelial cells in the proliferative endometrium. **b**, UMAP projections of epithelial nuclei (*n* = 105,095) from all individuals (*n* = 27) revealing 6 subclusters. **c**, Heatmap of scaled log(transformed) gene expression characterizing the six epithelial subclusters. **d**, Bar plot showing the proportion of the six epithelial subclusters from the control group (*n* = 5), the PCOS group (*n* = 12), the PCOS-metformin group (*n* = 7) and the PCOS-lifestyle group (*n* = 3). **e**, Violin plots of log(transformed) *ESR1* expression in the control, PCOS, PCOS-metformin and PCOS-lifestyle groups in AR^+^, SOX9^+^LGR5^+^and SOX9^+^ cycling epithelium cell types. The line inside the box indicates the median value and the box delimits the 25th and 75th percentiles. The smallest and largest values are indicated by the lines outside the box. ^***^BH-adjusted *P* < 0.001 in DEG analysis. **f**, Representative fluorescence microscopy images of fixed endometrial tissue from one PCOS and one control stained with EPCAM, a marker for glandular epithelial cells, cell adhesion and organoid membrane integrity, ESR1, DAPI and a merged image. **g**, Total number of DEGs in each epithelial subcluster and dotplots of the five most upregulated or downregulated DEGs (control versus PCOS) in each subcluster. **h**, GO enrichment analyses of DEGs divided by up- or downregulated genes within each epithelial subcluster between women with and women without PCOS at baseline and the changes from baseline to after 16 weeks of metformin or lifestyle management in women with PCOS. GOs with BH-adjusted *P* < 0.05 are reported and −log_10_(transformed) for visualization. **i**, Heatmap showing DEGs in GOs in epithelial subclusters between controls and PCOS and DEGs reversed by metformin or lifestyle intervention. **j**, Spatial transcriptomic with Stereo-seq of selected cell-type gene markers in epithelial subclusters of two control, two PCOS and one PCOS after 16 weeks of metformin and one PCOS after lifestyle management. See ‘Statistical analyses’ for a detailed description of DEG analysis and GO enrichment analysis statistics. Schematic in **a** created using BioRender.com. Source data

**Extended Data Fig. 3:**
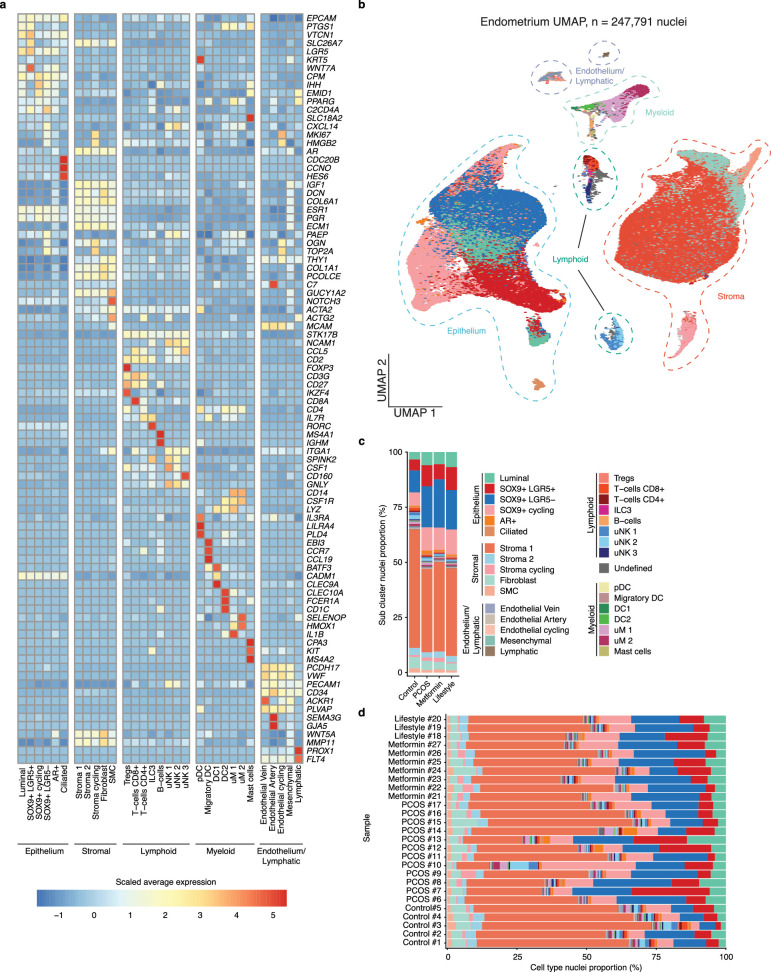
snRNA-seq heatmap of all marker genes and detailed UMAP. **a)** Heatmap of all gene markers used to identify cellular subpopulations on the Y-axis and corresponding subpopulations on X-axis. The color indicates the scaled averaged gene expression. **b)** Uniform manifold approximation and Projections (UMAP) of 247,791 integrated nuclei from 27 samples, annotated for identified cellular subpopulations. **c)** Bar plot of the proportion of identified cellular subpopulations per group; Control, n = 5; PCOS, n = 12, and after 16 weeks of intervention; PCOS-Metformin, n = 7, and PCOS-Lifestyle, n = 3. **d)** Bar plot of identified cellular subpopulations nuclei proportions of 27 samples.

**Extended Data Fig. 4:**
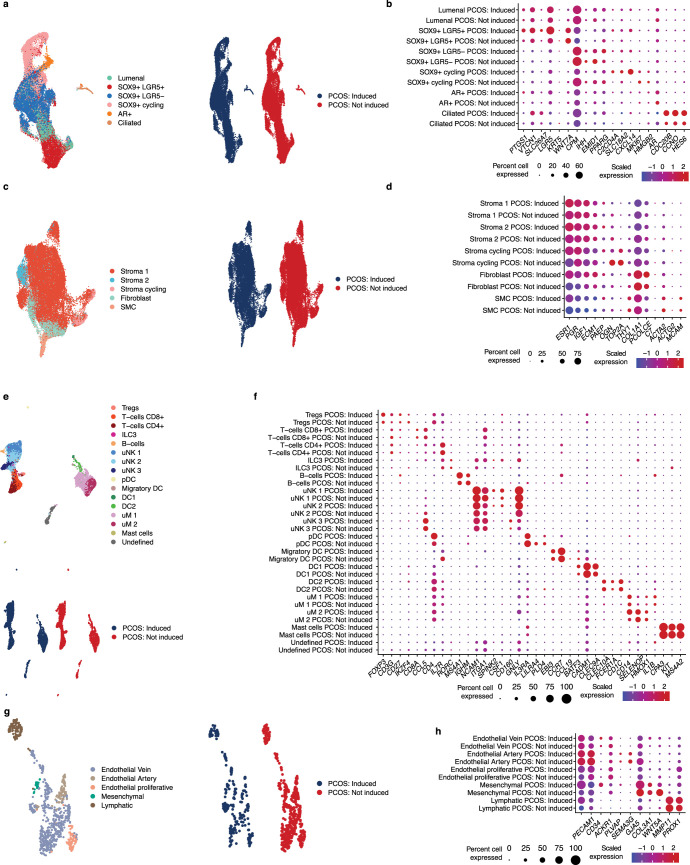
Subcluster analysis of induced versus non-induced bleeding samples. **a**) Uniform Manifold Approximation and Projection (UMAP) projections of epithelial nuclei from women with PCOS and Provera-induced (n = 5) and non-induced (n = 7) bleedings reveal six subclusters. **b**) Dotplots scaled log-transformed gene expression of cell type markers characterizing each epithelial subcluster. **c**) UMAP projections of stromal nuclei from women with PCOS and Provera-induced (n = 5) and non-induced (n = 7) bleedings reveal five subclusters. **d)** Dotplots show scaled log-transformed gene expression of cell type markers characterizing each stromal subcluster. **e**) UMAP projections of nuclei from women with PCOS and Provera-induced (n = 5) and non-induced (n = 7) bleedings of immune subclusters. **f**) Dotplots show scaled log-transformed gene expression of cell type markers characterizing each immune subcluster. **g)** UMAP projections of nuclei from women with PCOS and Provera-induced (n = 5) and non-induced (n = 7) bleedings of endothelial subclusters, and **h)** Dotplots show scaled log-transformed gene expression of cell type markers characterizing each endothelial subcluster.

**Extended Data Fig. 5:**
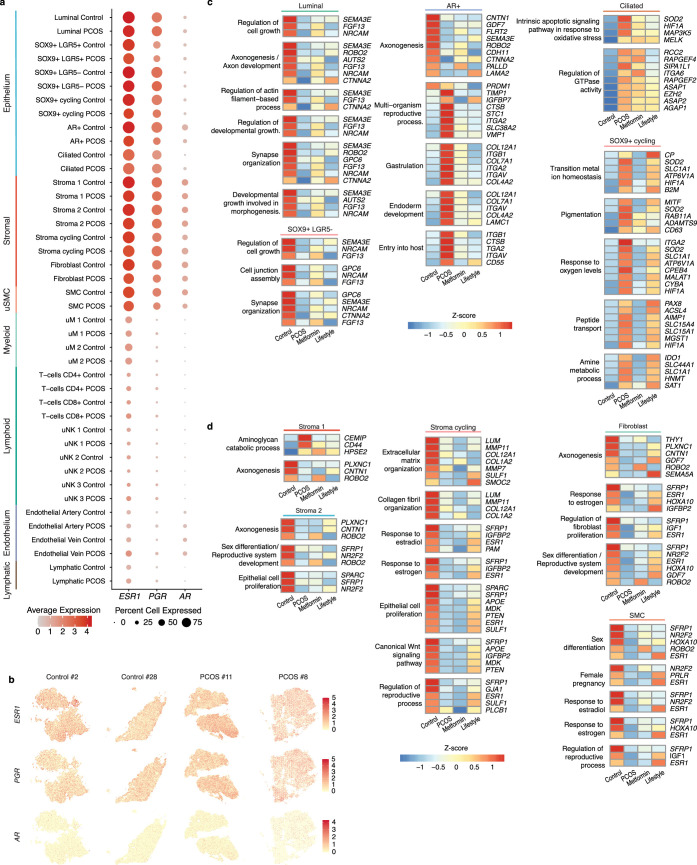
Expression levels of steroid receptors and Gene Ontology of significant signaling pathways PCOS versus controls and those reversed by intervention. **a)** Dotplot of snRNA-seq expression levels of *ESR1, PGR* and *AR* in each subcluster. **b**) Spatial transcriptomic distribution and gene expression of *ESR1, PGR* and *AR* in 2 control and 2 PCOS endometrial samples. The color bar indicated the gene expression level. **c**) Heatmap showing DEGs in GOs in the remaining epithelial subclusters between controls and PCOS and DEGs reversed by metformin or lifestyle intervention. **d**) Heatmap showing DEGs in GOs in the remaining stromal subclusters between controls and PCOS and DEGs reversed by metformin or lifestyle intervention.

Subcluster analysis of epithelium identified six subpopulations: (1) luminal positive, (2) SOX9^+^LGR5^+^, (3) SOX9^+^LGR5^−^, (4) SOX9^+^ cycling, (5) ciliated cells and (6) AR^+^ cells (Fig. 2a–c and Extended Data Fig. 3a–d), with no difference between samples obtained from women who had induced or noninduced bleeding (Extended Data Fig. 4a,b). Despite a higher proportion of epithelial nuclei in the PCOS-endometrium (Fig. 1e,f), there was no difference in the proportions among the epithelial subpopulations compared with control endometrium (Fig. 2d). Estrogen-α (*ESR1*), progesterone (*PGR*) and androgen (AR) receptors are critical for endometrial proliferation and differentiation, with expression varying during the menstrual cycle^9,17^. *ESR1* was downregulated in AR^+^, SOX9^+^LGR5^+^ and SOX9^+^ cycling epithelial subpopulations in women with PCOS versus women without the syndrome (Fig. 2e), confirmed at the protein level (Fig. 2f and Extended Data Fig. 1e) and consistent with a previous study^9^. This was partially reversed by metformin (Fig. 2e). Neither *AR* nor *PGR* (encoding PR) expression varied significantly, reflecting the proliferative phase. The dotplot and Stereo-seq illustrate *ESR1, PGR* and *AR* expression levels across subpopulations (Extended Data Fig. 5a,b).

Differentially expressed gene (DEG) analyses comparing samples from women with and women without PCOS in epithelial subpopulations identified the most DEGs in SOX9^+^LGR5^+^ (*n* = 138), SOX9^+^ cycling (*n* = 174), AR^+^ (*n* = 257) and ciliated (*n* = 259) subclusters. The five most up- and downregulated genes (Fig. 2g) and all DEGs per subcluster are listed in Supplementary Table 2a–c. Many genes are differentially expressed in multiple subclusters, suggesting a PCOS-specific composition of and effects on epithelial cells. For example, the expression of *SEMA3*, a semaphorin related to angiogenesis and cell proliferation, is increased in the proliferative phase compared with the secretory phase in non-PCOS and in participants with thin endometrium^18^. In the present study, *SEMA3E* is downregulated in all epithelial subclusters in women with PCOS, except ciliated cells. The *ROBO2* receptor is elevated in the proliferative phase and lower in the secretory phase in the non-PCOS-endometrium^19^, whereas it is downregulated in AR^+^, SOX9^+^LGR5^+^ and luminal subclusters in the PCOS-endometrium. PAEP, the progestin-associated endometrial protein, linked to recurrent pregnancy loss^20^, was upregulated in PCOS luminal, SOX9^+^LGR5^+^, SOX9^+^LGR5^−^ and AR^+^ subclusters. *NEAT1*, linked to endometrial cancer^21^, implantation failure and inflammation^22^, was upregulated in PCOS SOX9^+^LGR5^−^ and AR^+^ subclusters (Fig. 2g). *SLPI*, an antibacterial gene^23^, was upregulated in PCOS luminal, SOX9^+^LGR5^+^ and SOX9^+^LGR5^−^ subpopulations (Fig. 2g). *ITGA2*, associated with PCOS^24^, was specifically increased in SOX9^+^LGR5^+^ subpopulations (Fig. 2g). These findings suggest a distinct epithelial composition and transcriptomic profile in the endometrium of women with PCOS.

Gene ontology (GO) analysis of DEGs in PCOS versus controls revealed enriched pathways related to pregnancy, ovulation, endoderm development, tissue structure, integrin-mediated adhesion, signaling and extracellular matrix (ECM) organization across subpopulations (Fig. 2h, Extended Data Fig. 5c and Supplementary Table 3a–c). Metformin reversed signaling pathways and DEGs in SOX9^+^LGR5^+^ and AR^+^ subpopulations, whereas lifestyle management affected only AR^+^ subpopulations (Fig. 2i). In the ovulation cycle pathway of a PCOS-SOX9^+^LGR5^+^ subpopulation, metformin restored *ROBO2, MDK* (linked to endometrial cancer)^25^ and *ESR1* and *MAP2K6* (associated with recurrent implantation failure)^26^. For integrin-mediated signaling and ECM organization, in PCOS-endometrium both treatments restored expression of ArgGluAsp receptors (*ITGB6, ITGB8* and *ITGAV*), collagen receptor *ITGA2*, laminin receptor *ITGA3* and *ADAMTS9* (linked to reduced oocyte maturation in PCOS)^27,28^. Integrins, as transmembrane receptors, mediate ECM–cytoskeleton connections, transmitting critical biochemical and mechanical signals in disease^27^.

**Extended Data Fig. 6:**
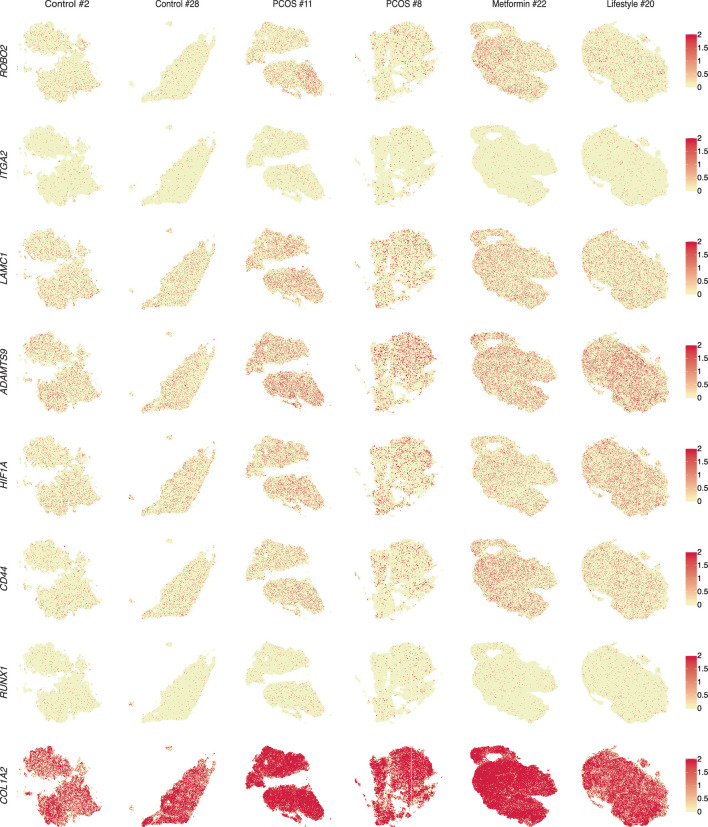
Spatial transcriptomic of selected DEGs. Spatial distribution and gene expression of selected DEGs from the CellChat analysis in Fig. 6; *ROBO2, ITGA2, LAMC1, ADAMTS9, HIF1A, CD44, RUNX1* and *COL1A2*. The color bar indicates the gene expression level.

To map the spatial distributions of subpopulations identified by snRNA-seq, we projected cell-type-specific marker genes on to Stereo-seq data, validating subpopulations and defining spatial coordinates (Fig. 2j). Luminal cells (*LGR5*), SOX9^+^LGR5^+^ (*WNT7A*), SOX9^+^LGR5^−^ (*IHH*), SOX9^+^ cycling (*PAEP*) and AR^+^ cells (*AR*) were mapped. Key markers related to endometrial dysfunction, such as *ROBO2*, *ITGA2* and *ADAMTS9*, were also visualized (Extended Data Fig. 6).

Overall, we characterized and localized epithelial subpopulations in the PCOS-affected endometrium, revealing increased proportions of epithelial cells with distinct DEG patterns linked to endometrial cancer, endometrial receptivity and implantation failure, supporting the link to PCOS-specific endometrial dysfunction in the proliferative phase. SOX9^+^LGR5^+^ and AR^+^ subpopulations exhibit enriched pathways in ECM organization, collagen metabolism, integrin signaling and pregnancy. These subpopulations are unique in their responsiveness to metformin and reverse several of the dysregulated genes. Lifestyle changes also contributed to gene regulation, although to a lesser extent.

### Spatiotemporal alterations in PCOS endometrial stromal cells

**Fig. 3:**
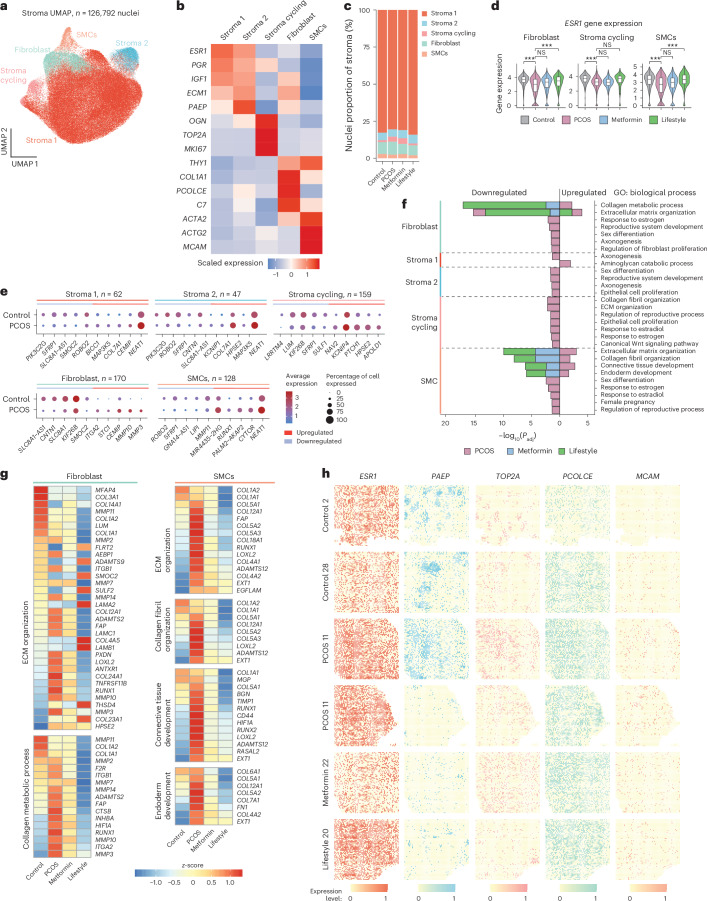
Temporal and spatial characterization of endometrial stromal cells and effect of metformin and lifestyle in women with PCOS. **a**, UMAP projections of the stromal nuclei subclusters (*n* = 126,792) from all individuals (*n* = 27). **b**, Heatmap of the scaled log(transformed) gene expression characterizing the five stromal subclusters. **c**, Bar plot showing the proportion of the five stromal subclusters from: control (*n* = 5), PCOS (*n* = 12), PCOS-metformin (*n* = 7) and PCOS-lifestyle (*n* = 3). **d**, Violin plots of log(normalized) *ESR1* expression in the control, PCOS, PCOS-metformin and PCOS-lifestyle groups in fibroblast, stroma cycling and SMC cell types. The line inside the box indicates the median value and the box delimits the 25th and 75th percentiles. The smallest and largest values are indicated by the lines outside the box. ^***^BH-adjusted *P* < 0.001 in DEG analysis**. e**, Total number of DEGs in each stromal subcluster and dotplots of the top five up- and downregulated DEGs (control versus PCOS) in each subcluster. **f**, GO enrichment analyses for DEGs split on up- or downregulation, within each stromal subcluster at baseline between control and PCOS and between PCOS-baseline and after 16 weeks of intervention with metformin or lifestyle, respectively. GOs with BH FDR *P* < 0.05 are reported and −log_10_(transformed) for visualization. **g**, Heatmap showing DEGs in GOs in stromal subclusters between controls and PCOS and DEGs that are reversed by metformin or lifestyle intervention. **h**, Spatial transcriptomic with Stereo-seq of selected cell-type markers in stromal subclusters. See ‘Statistical analyses’ for a detailed description of DEG analysis and GO enrichment analysis statistics. Source data

Stromal cells were annotated into five subpopulations: (1) stroma 1, (2) stroma 2, (3) proliferative stroma, (4) fibroblasts and (5) SMCs (Fig. 3a,b and Extended Data Fig. 3a–d) with no difference between induced and noninduced bleeding samples (Extended Data Fig. 4c,d). Although the proportion of stromal cells was lower in PCOS-endometrium compared with non-PCOS-endometrium, subpopulation proportions remained unchanged after intervention (Fig. 3c).

As in the epithelium, *ESR1* was downregulated in the PCOS-endometrium compared with the non-PCOS-endometrium in proliferative stromal cells, fibroblasts and SMCs, and is reversed by lifestyle management in women with PCOS (Fig. 3d). Extended Data Fig. 5a,b shows the expression level of *ESR1, PGR* and *AR* in all stromal subpopulations in controls and cases of PCOS with no change after intervention. The comparison between women with and women without PCOS revealed the highest number of DEGs in proliferative stroma (*n* = 159), fibroblasts (*n* = 170) and SMCs (*n* = 128). Key up- and downregulated DEGs in PCOS in stroma 1 and stroma 2, which were not differentially expressed in any other subpopulation, included *PIK3C2G* (downregulated), a gene associated with granulosa cell tumor^29^, *MAP3K5* (upregulated), a marker of human endometrial receptivity^30^, and the collagen gene *COL7A1* (upregulated), a component of the basement membrane and surrounding vasculature that plays an important role in regulating tissue remodeling and homeostasis^31^ (Fig. 3e and Supplementary Table 2a–c). In addition, *SFRP1*, a Wnt signaling inhibitor with higher expression in the proliferative phase^32^, is downregulated in the stroma 1, stroma 2, stroma cycling and SMC subpopulation in women with PCOS compared with controls. Moreover, *NEAT1* is also upregulated in stroma 1, stroma 2 and SMCs in the PCOS-endometrium.

GO for biological processes of DEGs between PCOS cases and controls revealed enriched pathways related to estrogen response, reproductive development, epithelial organization and ECM and collagen organization, particularly in fibroblasts, proliferative stroma and SMCs (Fig. 3f). In women with PCOS, metformin and lifestyle management (to a lesser extent) reversed dysregulated collagen metabolism, ECM organization and connective tissue development exclusively in fibroblasts and SMCs (Fig. 3f,g, Extended Data Fig. 5d and Supplementary Table 3a–c). In fibroblasts, lifestyle and, to lesser extent, metformin normalized elevated *HIF1A* (impairing stromal fibroblast decidualization critical for embryo implantation)^33^, *RUNX1* (linked to endometrial cancer and angiogenesis)^34^, *MMP3* and *MMP10* (involved in extracellular remodeling and associated with endometriosis)^35^ and *ITGA2*, often upregulated in endometrial cancer and PCOS epithelial cells^24^. In SMCs, both treatments restored collagen-encoding genes and the expression of fibronectin and *CD44*.

Next, we projected the cell-type-specific stromal cell marker genes from snRNA-seq on to the Stereo-seq data to validate each subpopulation and define specific spatial coordinates for the specific marker genes: *ESR1* (stroma 1), *PAEP* (stroma 2), *TOP2A* (cycling stroma), *PCOLCE* (fibroblasts) and *MCAM* (SMCs) (Fig. 3h). *HIF1A*, *RUNX1*, *ITGA2* and *CD44* were also visualized (Extended Data Fig. 6).

In summary, we defined and spatially localized the stromal subpopulations in the PCOS-endometrium and identified a reduced proportion of stromal cells, suggesting slower proliferation. We observed distinct DEG patterns associated with endometrial cancer, as well as endometrial receptivity and implantation failure. Metformin and lifestyle management restored pathways associated with collagen metabolism and ECM organization, particularly in fibroblasts and SMCs, reflecting treatment-specific improvements in PCOS-specific endometrial dysfunction.

### PCOS endometrial immune and endothelial cell alterations

**Fig. 4:**
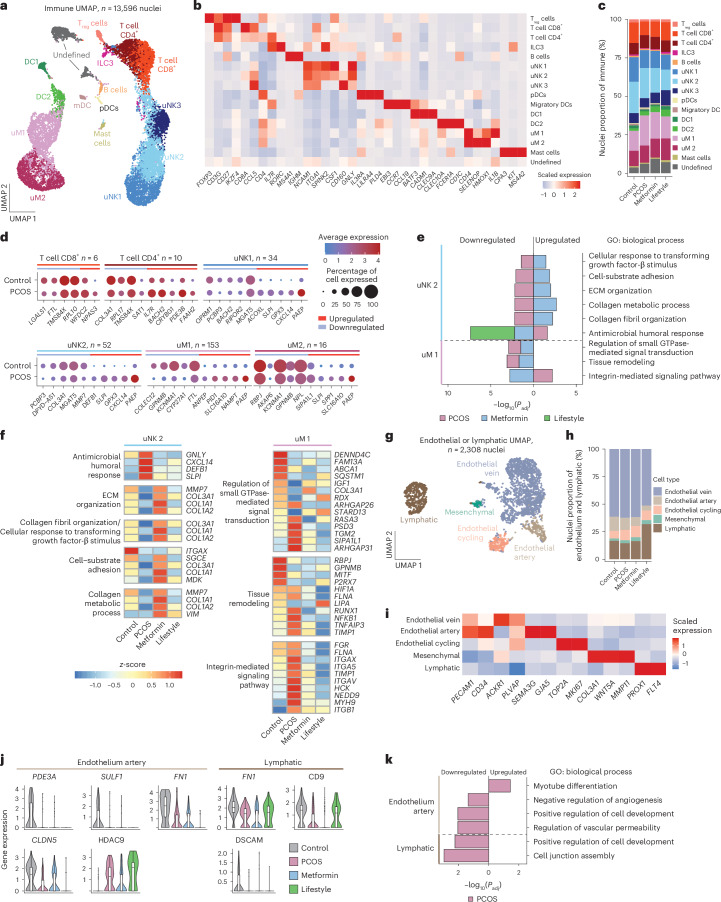
Immune cell subcluster in the PCOS-endometrium and effect of metformin and lifestyle. **a**, UMAP of subclustered immune cells (*n* = 13,596) from all individuals (*n* = 27). **b**, Heatmap of scaled log(transformed) gene expression characterizing 15 immune subclusters. **c**, Bar plot showing the proportion of the 15 immune subclusters from control (*n* = 5), PCOS (*n* = 12), PCOS-metformin (*n* = 7) and PCOS-lifestyle (*n* = 3) groups. **d**, Total number of DEGs in the six immune subclusters and dotplots of the top five up- and downregulated DEGs (control versus PCOS) in these immune subclusters. **e**, GO enrichment analyses on DEGs split on up- or downregulation, within six immune subclusters at baseline between PCOS and controls, and between PCOS-baseline and after 16 weeks of intervention with metformin or lifestyle, respectively. GOs with BH-adjusted *P* < 0.05 are reported and −log_10_(transformed) for visualization. **f**, Heatmap showing DEGs in GOs between controls and cases of PCOS and those that are reversed by metformin or lifestyle intervention. **g**, UMAP of subclustered endothelial or lymphatic cells from all individuals. **h**, Bar plot showing the proportion of the five endothelial or lymphatic subclusters from control (*n* = 5), PCOS (*n* = 12), PCOS-metformin (*n* = 7) and PCOS-lifestyle (*n* = 3) cases. **i**, Heatmap of scaled log(transformed) gene expression characterizing the five endothelial or lymphatic subclusters. **j**, Violin plot showing DEGs in GOs between PCOS cases and controls, and between PCOS-baseline and after 16 weeks of intervention with metformin or lifestyle, respectively. The line inside the box indicates the median value and the box delimits the 25th and 75th percentiles. The smallest and largest values are indicated by the lines outside the box. **k**, GO enrichment analyses on DEGs split on up- or downregulation, within endothelial artery and lymphatic subclusters at baseline between control and PCOS cases and between PCOS-baseline and after 16 weeks of intervention with metformin or lifestyle, respectively. GOs with BH FDR *P* < 0.05 are reported and −log_10_(transformed) for visualization. See ‘Statistical analyses’ for a detailed description of DEG analysis and GO enrichment analysis statistics. Source data

PCOS is linked to low-grade inflammation^3^ and a dysfunctional immune microenvironment, which is, in turn, associated with impaired endometrial receptivity^5,10,36^. In a mouse model of PCOS, hyperandrogenism increased uterine natural killer (uNK) cell frequency, an effect that was reversed by AR antagonists^37^. We characterized 13,596 endometrial immune cells, identifying three uNK populations (uNK1, uNK2 and uNK3), two uterine macrophage populations (uM1 and uM2), regulatory T cells, CD8^+^ and CD4^+^ T cells, B cells, dendritic cells (DC1 and DC2) and mast cells. Despite these findings, immune subpopulation proportions did not differ between women with and women without PCOS (Fig. 4a,c and Extended Data Fig. 3a–d).

The largest immune subpopulations with the most DEGs were uNK1 (*n* = 34), uNK2 (*n* = 52) and uM1 (*n* = 153) (Fig. 4d and Supplementary Table 2a–c), with no difference between induced and noninduced bleeding samples (Extended Data Fig. 4e,f). These cells, active during the menstrual and proliferative phases, are crucial for wound healing and endometrial regrowth, processes impaired in PCOS^38^. Key genes elevated in uNK1, uNK2 and uM2 include *GPX3*, linked to embryo quality^39^, and *SLPI*, *CXCL14*, *PAEP* and *NAMPT*, important for endometrial receptivity^13,36^ (Fig. 4d). Remarkably, metformin reversed *PAEP* expression and partially by lifestyle (Extended Data Fig. 2c). Moreover, *PAEP* expression was projected to immune annotated clusters in the Stereo-seq data, confirming its expression (Extended Data Fig. 2d).

GO analysis revealed pathway enrichment in uNK2 and uM1, with most uNK2 pathways downregulated in PCOS but restored after 16 weeks of metformin, but to a less extent by lifestyle (Fig. 4e,f and Supplementary Table 3a–c). Metformin also normalized ECM- and collagen-related genes (*COL1A1*, *COL1A2*, *COL3A1* and *MMP7*) in uNK2 and integrin-mediated pathways in uM2 (Fig. 4e,f).

In the PCOS-endometrium, the smallest subcluster, endothelial or lymphatic (2,308 nuclei) (Fig. 4g–i), showed downregulation of *PDE3A*, *SULF1*, *FN1* and *CLDN5* in an endothelial artery subcluster and *FN1*, *CD9* and *DSCAM* in lymphatic cells, with upregulation of *HDAC9* in the endothelial artery subcluster (Fig. 4j, Supplementary Table 2a–c and Extended Data Fig. 4g,h). These changes were unaffected by interventions. GO analysis highlights pathways in cell development and junction assembly in lymphatic cells (Fig. 4k and Supplementary Table 3a–c).

Among immune subpopulations, uNK1, uNK2 and uM1 were most impacted in the PCOS-endometrium. Dysregulated pathways involve collagen metabolism, ECM organization and integrin signaling. ECM- and collagen-related genes are downregulated in uNK2, whereas integrin signaling genes are upregulated in uM1, with both responsive to metformin treatment.

### Cell–cell interactions in the PCOS-endometrium

SnRNA-seq and Stereo-seq data reveal distinct dysregulated genes and biological processes in epithelial, stromal and immune subpopulations of the PCOS-endometrium. Intercellular communication within and between subpopulations is critical for endometrial response to hormonal imbalance^16^. Using CellChat, we analyzed cell-to-cell ligand–receptor interactions among subpopulations. The interaction probability, signaling pathways and ligand–receptor pairs are detailed in Supplementary Table 4a–e.

**Extended Data Fig. 7:**
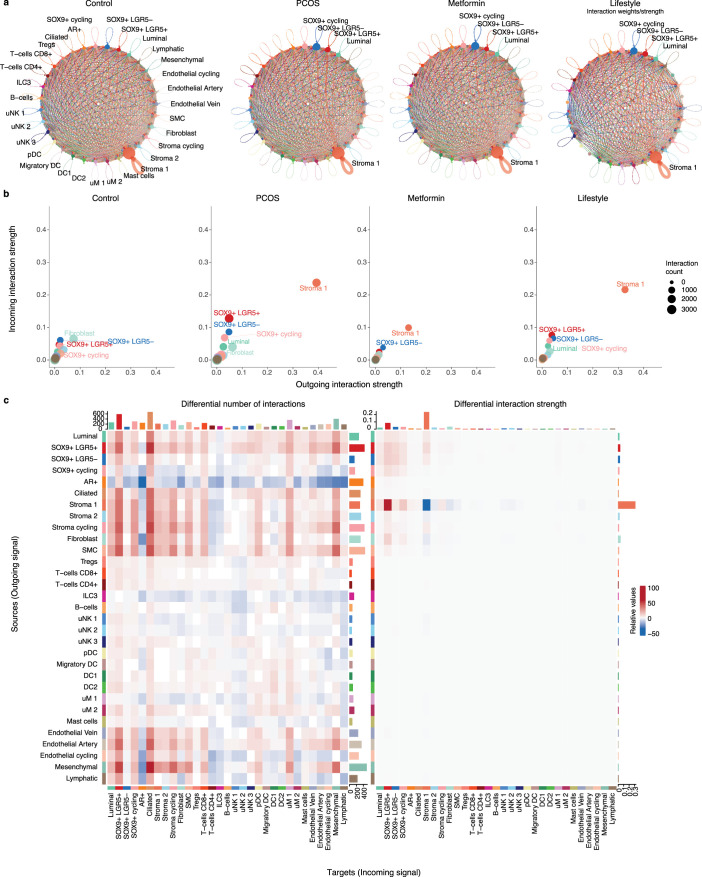
CellChat cell-cell interactions. **a)** Interaction diagram of all identified subclusters with interaction strengths, where the line thickness indicates the interaction strength**. b)** Scatter plots with the incoming interaction strength (receptor) on the Y-axis and the outgoing interaction strength (ligand) on the X-axis of each subcluster per group. **c)** Heatmaps of the different number of interactions and interaction strengths when comparing PCOS with the control. The colored bars at the top show the cumulative value of each column of incoming signals in each subcluster. The colored bar graph on the right shows the cumulative value of the outgoing signals in each subcluster. The height of the bar indicates the difference in the number and strength of interactions between the groups. The colored bars indicate whether the signal transmission between the subclusters is increased (red) or decreased (blue) in PCOS compared to the control group.

In the PCOS-endometrium, the stroma 1 subpopulation exhibited dominant interaction strength with SOX9^+^ cycling, SOX9^+^LGR5^+^, SOX9^+^LGR5^−^ and luminal subpopulations. These interactions were unaffected by metformin and lifestyle interventions (Extended Data Fig. 7a–c).

**Fig. 5:**
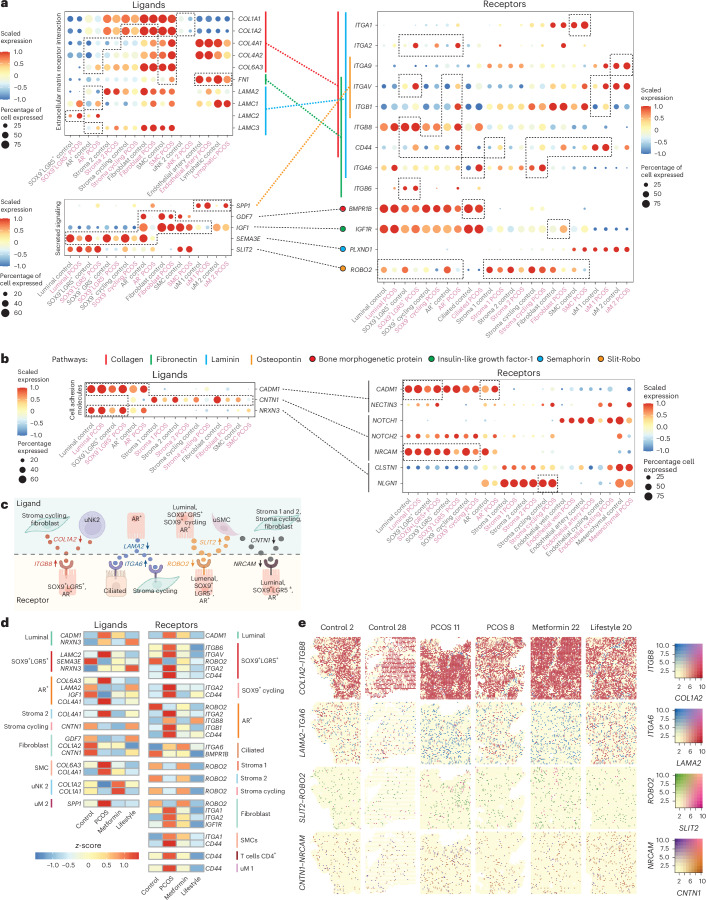
Distinct epithelial–stromal immune cell crosstalk and ligand–receptor interaction. Dotplots of genes encoding ligands and receptors of CellChat-predicted interactions and signaling pathways in different cell subpopulations. **a**, The ligand dotplots refering to signaling pathways annotated as ECM–receptor interaction or associated secreted signaling. The dashed and filled lines indicate which ligand–receptor pars interact on the *y* axis. The colors are explained in the pathway legend. The dashed boxes indicate DEGs (MAST model in Seurat) and cell subpopulations on the *x* axis. All dots are scaled on the average log(transformed) expression indicated by the color and the size of the dot shows the percentage of cells expressing the gene. **b**, Dotplot of genes encoding ligand–receptor pairs annotated as cell adhesion molecules by CellChat. **c**, Illustration of selected cell subpopulation and specific receptors and ligands. The arrows indicate whether the encoding genes are up- or downregulated in PCOS. **d**, Heatmap of genes encoding ligands and receptors that are reversed after metformin or lifestyle treatment compared with PCOS. The colored bars on the side of the heatmap indicate to which cell subpopulations the genes belong. The colors of the blocks in the heatmap represent the *z*-score, that is, a scaled average of log(transformed) gene expression. **e**, Spatial distribution and gene expression of selected interacting ligand and receptor genes from the CellChat analysis in Fig. 6a,b. The color bar indicates the coexpression level of the genes. Schematic in **c** created using BioRender.com.

**Extended Data Fig. 8:**
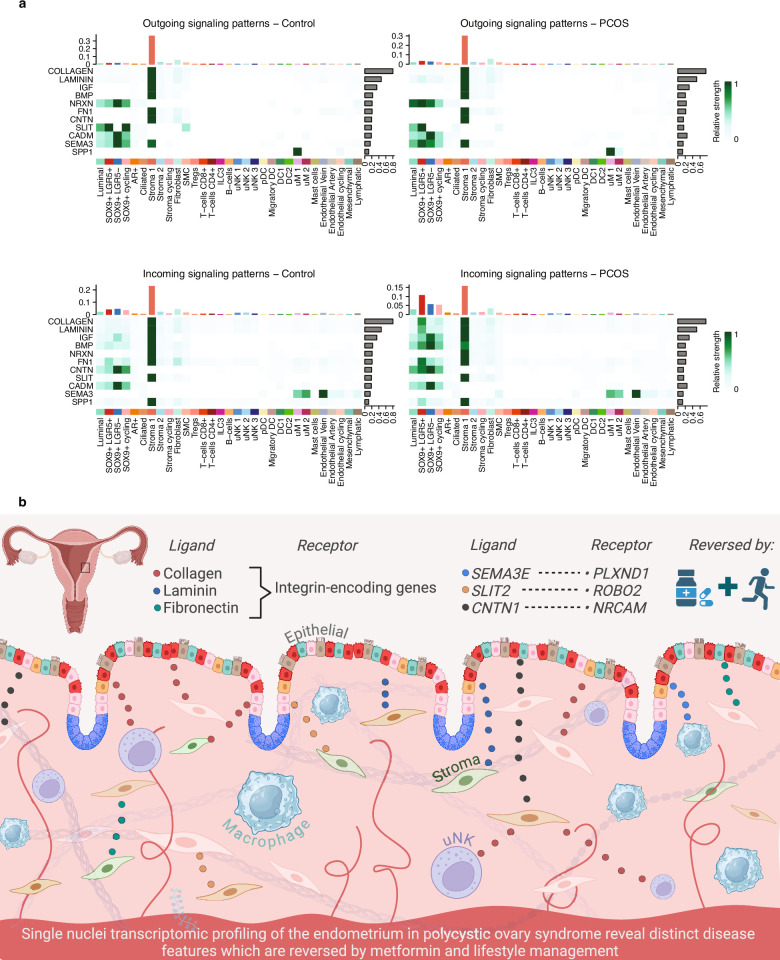
CellChat signaling heatmap of selected outgoing and incoming pathways in PCOS and controls. **a**) The colors of the heatmap show the relative strength and represent the strength of the pathway per subcluster. The bar graphs on the top and right axis show the cumulative strength of the pathway and each subcluster. **b**) Graphical illustration summarizing the key message. Illustration in **b** created using BioRender.com.

Next, analysis of ligand–receptor genes in the different subpopulations revealed that the strongest interactions in the PCOS-endometrium involved ECM components, including collagen, laminin and fibronectin 1 (FN1) pathways, aligning with DEGs in major subpopulations (Fig. 5a,b, Extended Data Fig. 8a and Supplementary Table 4a–e).

Key findings in the PCOS-endometrium include downregulation of collagen ligands *COL1A1* in uNK2 and *COL1A2* in proliferative stroma, fibroblasts and uNK2, whereas *COL4A1*, *COL4A2* and *COL6A3* are upregulated in AR^+^ epithelium, stroma 2 and SMCs (Fig. 5a and Supplementary Table 4a–e). Collagen ligands interact with upregulated integrin-encoding genes, such as *ITGA1* in SMCs, *ITGA2* in SOX9^+^LGR5^+^, SOX9^+^ cycling and AR^+^ and *ITGAV* and *ITGB1* in AR^+^ and uM1. *CD44* was expressed across epithelial and stromal subclusters, except luminal, ciliated, stroma 2 and uM2 (Fig. 5a,d and Supplementary Table 4a–e). This interaction supports the activation of ECM signaling pathways, potentially driving the increased epithelial cell proportion in the PCOS-endometrium through enhanced proliferation.

*FN1* is downregulated in SMCs, endothelial arterial and lymphatic cells, whereas *LAMA2* is downregulated in AR^+^ epithelial cells. In contrast, *LAMC1* and *LAMC3* are upregulated in AR^+^ cells and *LAMC2* in SOX9^+^LGR5^+^ cells. These *FN1* and laminin ligands interact with the same integrin-encoding genes as collagen ligands (Fig. 5a and Supplementary Table 4a–e).

In the secretory signaling pathways, *SPP1* (osteopontin) is upregulated in uM1 and uM2 in the PCOS-endometrium, forming ligand–receptor pairs with *ITGA9* (upregulated in uM2), *ITGAV* (in SOX9^+^LGR5^+^, AR^+^ and uM1) and *ITGB1* (in AR^+^ and uM1) (Fig. 5b and Supplementary Table 4a–e). *GDF7* is downregulated in AR^+^ and fibroblast cells, interacting with the *BMPR1B* receptor, which is downregulated in ciliated cells. *IGF1* is downregulated in AR^+^, fibroblasts, SMCs and uM1 cells, interacting with *IGF1R*. *SEMA3E*, downregulated across epithelial subpopulations, interacts with *PLXND1*, the expression of which remains unchanged. In the Slit-Robo pathway, only the *ROBO2* receptor is downregulated in epithelial and stromal subpopulations (Fig. 5b–d and Supplementary Table 4a–e).

Cell adhesion molecule-related signaling pathways including *CADM1*, acting as both ligand and receptor, are upregulated in luminal, SOX9^+^LGR5^+^ and AR^+^ epithelial cells and interact with *NECTIN3*, which is not differentially expressed (Fig. 5c and Supplementary Table 4a–e). *CNTN1*, downregulated in AR^+^ and stromal subpopulations, interacts with *NOTCH1-2* and *NRCAM* receptors, the latter showing differential expression in epithelial subpopulations except AR^+^ (Fig. 5c,d). *NRXN3*, upregulated in luminal and SOX9^+^LGR5^+^, interacts with *CLSTN1* and *NLGN1*, with the latter elevated in proliferative stroma in PCOS.

Importantly, many DEGs encoding ligands and receptors are among the top-ranked genes in PCOS versus controls (Figs. 2–4). Comparison of CellChat DEGs with treatment-responsive genes revealed significant changes in collagen, integrin, *CD44* and *ROBO2* expression, which was further validated by Stereo-seq data (Fig. 5d,e and Extended Data Fig. 6).

Our analysis uncovers key dysregulations on integrin receptors activated by collagen, fibronectin 1 and laminin in SOX9^+^LGR5^+^, AR^+^ epithelial cells and uM1 and uM2 subpopulations in the PCOS-endometrium. Despite their key role in menstrual endometrium^40^, integrins in PCOS remain underexplored. These adhesion molecules, capable of bidirectional signaling in response to extracellular changes, may represent therapeutic targets for endometrial dysfunction^27^ (Extended Data Fig. 8b).

### Endometrial cell types and human diseases

PCOS is associated with a variety of reproductive and metabolic diseases^3^. Genome-wide association studies (GWASs) have suggested a link between PCOS and bioavailable testosterone, endometrial cancer and endometriosis^41^. In addition, PCOS shares genetic loci with T2D, fasting insulin and glucose, as well as BMI-adjusted waist:hip ratio (WHR)^42^. Using CELLEX and CELLECT, we integrated snRNA-seq data with GWASs to identify endometrial cell types mediating these associations.

**Fig. 6:**
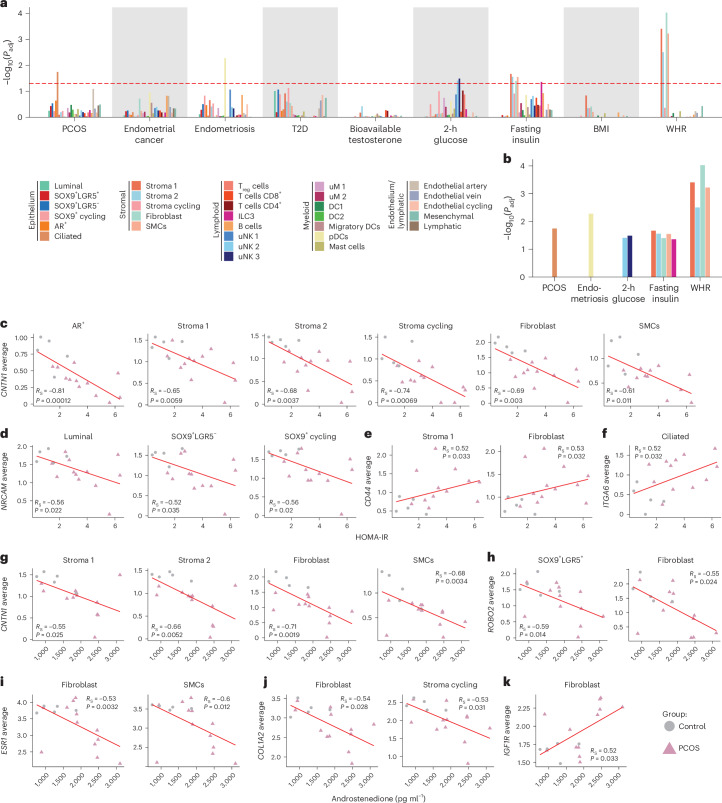
Subcluster–disease associations via GWASs and DEG correlations with insulin resistance and hyperandrogenemia. **a**, CELLECT analysis of the association between cell types in human endometrial snRNA-seq data (PCOS + controls) with GWASs. The red dashed line outlines the −log_10_(transformed) Bonferroni’s adjusted *P* = 0.05. The colors of the bars indicate the subclusters listed in the legend. Fasting insulin, 2-h glucose, T2D and WHR were BMI adjusted. Subclusters with a higher adjusted −log_10_(*P*) than the red dashed line indicate significant enrichment. **b**, A magnification of GWASs and subclusters identified as significantly enriched by CELLECT analysis. The exact adjusted *P* values (**a** and **b**) can be found in Supplementary Table 5b. **c**, Spearman’s correlation of averaged gene expression of DEGs per subcluster. *CNTNT1* is negatively correlated with HOMA-IR in epithelial and stromal subpopulations. **d**, *NRCAM1* negatively correlated with HOMA-IR in epithelial subpopulations. **e**,**f**, *CD44* (**e**) and *ITGA6* (**f**) positively correlated with HOMA-IR in stromal subpopulations. **g**, *CNTNT1* negatively correlated with androstenedione in stromal subpopulations. **h**, *ROBO2* negatively correlated with androstenedione in epithelial subpopulations. **i**,**j**, *ESR1* (**i**) and *COL1A2* (**j**) negatively correlated with androstenedione in stromal subpopulations. **k**, *IGFR1* positively correlated with androstenedione in stromal subpopulations. See ‘Statistical analyses’ for a detailed description of Spearman’s correlations. *R*_S_, Spearman’s rank correlation.

PCOS GWASs showed a significant association with ciliated epithelial cells, whereas endometriosis GWASs were linked to plasmacytoid dendritic cells (pDCs). Stromal subpopulations, except proliferative stroma, were strongly associated with BMI-adjusted WHR. In addition, uNK2 and uNK3 associated with 2-h glucose-level GWASs and fasting insulin GWASs were associated with stroma 1, stroma 2, fibroblasts, proliferative endothelial cells and type 3 innate lymphoid cells (ILC3 cells) (Fig. 6a,b and Supplementary Table 5a–d).

To investigate the relationship between calculated averaged gene expression of DEGs per cell type and PCOS clinical traits, correlation analyses were performed across cell types at baseline including all women (Supplementary Table 6). *CNTN1*, downregulated in AR^+^ and stromal subpopulations in PCOS, negatively correlated with HOMA-IR and androstenedione in stromal cells (Fig. 6c,g). *NRCAM*, the *CNTN1* receptor, inversely correlated with HOMA-IR in epithelial subpopulations (Fig. 6d). *CD44* and *ITGA6* showed positive correlations with HOMA-IR in stroma 1, fibroblasts and ciliated epithelial cells (Fig. 6e,f). *ROBO2*, *ESR1* and *COL1A2* inversely correlated with androstenedione in epithelial and stromal subpopulations, whereas *IGF1R* was positively associated in fibroblasts (Fig. 6h–k).

Together these findings suggest that hyperandrogenism and insulin resistance predominantly affect epithelial subclusters (AR^+^, SOX9^+^LGR5^+^, SOX9^+^LGR5^−^, SOX9^+^ cycling) and stromal subclusters (stroma 1, stroma 2, stroma cycling, fibroblasts), contributing to the risk of endometrial dysfunction in women with PCOS.

## Discussion

PCOS affects millions globally, reducing endometrial receptivity^43^, increasing miscarriage risk and elevating endometrial cancer susceptibility^3,44^. However, its cellular and molecular heterogeneity and treatment responses remain unclear. In the present study, we present the most comprehensive proliferative-phase endometrial atlas in overweight, hyperandrogenic, insulin-resistant women with PCOS and controls of similar age, weight and BMI, and post 16-week first-line treatment effects in PCOS by analyzing ~250,000 nuclei. We defined cell-type-specific molecular signatures and altered cellular composition and spatial localization of PCOS-specific cells. Distinct disease-specific signatures partially recover with metformin and lifestyle management. The present PCOS-endometrial cell atlas (PECA) study advances consensus-based cell annotation^13,14,16^ and provides a foundation for future single-cell PCOS endometrial research.

The increased endometrial disease risk in PCOS arises from altered sex steroid profiles, particularly hyperandrogenism^3^. Elevated testosterone leads to follicular arrest and chronic anovulation, causing unopposed estrogen exposure^3,4^, which promotes endometrial proliferation and elevates hyperplasia and cancer risk^45^. Insulin resistance and hyperinsulinemia further exacerbate this via direct mitogenic effects^3^. Obesity, affecting >50% of patients with PCOS, independently increases cancer risk^46^ by enhancing androgen production and peripheral estrogen conversion^47^. Aberrant endometrial gene expression suggests intrinsic dysfunctions linked to reduced receptivity, miscarriages and poor pregnancy outcomes^5,10,43,48,49^, although specific cellular contributions remain unclear.

The present study examined transcriptional and compositional differences in the proliferative PCOS and control endometrium. Patients with PCOS were hyperandrogenic, had elevated luteinizing hormone (LH) levels and were insulin resistant, with no age, weight or BMI differences from controls. Notably, the PCOS-endometrium had more epithelial cells and fewer stromal and lymphoid cells, despite synchronized biopsy collection and no evidence of hyperplasia. Although gene expression changed, these cellular differences persisted post 16-week metformin or lifestyle intervention. An increased epithelial:stromal ratio may contribute to the fivefold higher endometrial cancer risk in PCOS^50^.

We identified robust transcriptomic differences in PCOS endometrial, epithelial and stromal subclusters, with upregulated genes involved in cell adhesion, ECM and integrin signaling (*ITGAV*, *ITGA2*, *ITGA3*, *ITGB6*, *ITGB8*, *CD44* and *ADAMTS9*), particularly in SOX9^+^LGR5^+^ and AR^+^ subclusters. Notably, *ITGA2* and *ITGA3*, linked to endometrial cancer^24^, suggest integrin inhibitors as potential therapies^40^. These changes probably drive increased proliferation, reduced differentiation of epithelial cells and heightened cancer risk. The less differentiated epithelial phenotype in endometrial cancer and endometriosis, marked by SOX9^+^LGR5^+^ and SOX9^+^LGR5^−^ signatures^4^, aligns with our PCOS findings.

Sex hormone fluctuations regulate endometrial growth, degeneration and immune responses, shaping local immune populations. In the PCOS-endometrium, uNK2 and uM1 subpopulations are most affected, with elevated *PAEP*, linked to pregnancy loss and implantation failure^13,20^, and *SLPI*, *CXCL14* and *NAMPT* associated with endometrial cancer^13,23^. These findings highlight specific immune subpopulations as key players in PCOS pathophysiology and potential therapeutic targets.

Cell-type-specific differential gene expression correlates with clinical PCOS features, including HOMA-IR and androstenedione, suggesting that hyperandrogenism and insulin resistance contribute to endometrial dysfunction. GWASs suggest that epithelial cells influence PCOS risk, whereas stromal cells impact hyperinsulinemia and WHR. However, CELLECT findings require cautious interpretation because they identify cell populations enriching GWAS signals rather than effector genes^51^.

Ligand–receptor analysis, supported by Stereo-seq, reveals unique epithelial–stromal and uNK2 and uM1 immune subpopulation interactions in the PCOS-endometrium. In PCOS, epithelial subclusters activate COLLAGEN, LAMININ, cell adhesion molecule (CADM) and protein tyrosine phosphatase receptor type M (PTPRM) pathways, whereas stromal subclusters engage PTPR, insulin-like growth factor (IGF), adhesion G protein-coupled receptor L (ADGRL) and SLIT. In contrast, immune subclusters show downregulation of cholesterol, FN1, PTPRM, CD45 and COLLAGEN pathways. critical for ECM turnover, and mediate collagen uptake and degradation while producing collagens for tissue remodeling^52^, reflecting PCOS-related dysregulation.

Spatiotemporal single-nuclei analysis of the PCOS-endometrium reveals that 16 weeks of metformin and lifestyle management reverse many dysregulated genes, particularly in epithelial SOX9^+^LGR5^+^ and AR^+^ cells, stromal fibroblasts, SMCs and uNK2 and uM2 subpopulations. Metformin, a hydrophilic compound^53^, acts via mitochondria activating AMP-activated protein kinase (AMPK), mammalian target of rapamycin (mTOR) and GLUT4 to inhibit gluconeogenesis^54^. Beyond insulin sensitization, in vitro studies suggest antiproliferative and anti-metastatic effects in estrogen-dependent, endometrial carcinoma-like cells^55–57^. Notably, metformin reduced testosterone, androstenedione and free androgen index (FAI), but not HOMA-IR, suggesting that hyperandrogenism and/or hyperinsulinemia drive cell-specific molecular changes. These findings highlight metformin’s role in targeting integrin signaling and dysregulated pathways to improve endometrial health in PCOS.

Our data reveal a distinct disease-specific transcriptomic profile in epithelial, stromal, immune and endothelial subclusters of the PCOS-endometrium. Despite uniform proliferative-phase biopsies and similar age, weight and BMI between groups, large-scale profiling across all menstrual phases is needed. Notably, Provera-induced and noninduced bleeding showed no differences in nuclei proportions or subclustering.

This single-cell transcriptomic atlas of the PCOS-endometrium (PECA) provides a valuable resource for understanding PCOS-associated endometrial diseases, including cancer. The present study is unique in that it includes detailed and standardized phenotyping, which we link to the transcriptomic profile of specific endometrial cell types in the PCOS-endometrium. These results open new therapeutic targets, such as integrin inhibitors, and underscore the therapeutic role of metformin in modulating cell-type-specific endometrial dysfunction for endometrial health in women with PCOS.

## Methods

### Participants and interventions

The study adheres to good clinical practice and follows the Declaration of Helsinki. Approval has been granted by the Regional Ethical Review Board of Stockholm, Sweden (Dnr: 2015/1656-31/2 with amendment Dnr: 2024-00633-02) and is approved by the Medical Products Agency (EUCT: 2024-514505-64-00) and registered at Clinicaltrials.gov (NCT02647827). The women were recruited via online advertisements and examined at the Women’s Health Research Unit at the Karolinska University Hospital, Stockholm. Before any assessments, women received oral and written information and provided written informed consent. All women included in the study were aged ≥18 to ≤40 years with a BMI ≥ 25. PCOS diagnosis was set according to the revised 2003 Rotterdam criteria, excluding any endocrine-related disorder^58^. Transvaginal ultrasound measured the number of antral follicles (2–9 mm), ovarian volume (>10 cm^3^) and endometrial thickness (mm). Women with PCOS had irregular menstrual cycles >35 d. The Ferriman–Gallwey score was used to assess hirsutism with a score >4 considered as clinical hyperandrogenism. The control participants did not differ from PCOS in terms of age, weight and BMI. In addition, controls had <12 antral follicles (2–9 mm), an ovarian volume <10 cm^3^, regular menstrual cycles (±28 d) and a Ferriman–Gallwey score ≤4. Both women with and women without PCOS had no other gynecological diseases (for example, uterine fibroids, endometriosis), were neither pregnant nor breastfeeding in the last 6 months and were not taking any medication or hormonal treatment, including oral contraceptives, for at least 3 months before the baseline examinations, except for some women who took Provera 10 mg for 7 d to induce bleeding.

Serum analyses included sex steroids: DHEA, androstenedione, testosterone, estradiol and progesterone measured by gas chromatography–tandem mass spectroscopy^59^. All other hormones were analyzed at a Karolinska University Hospital-accredited laboratory by the following methods: LH and follicle-stimulating hormone (FSH) with electrochemiluminescence, Elecsys LH and FSH, respectively, and sex hormone-binding globulin by Roche Diagnostics analyzed on Cobas 8000 (Roche Diagnostics Scandinavia AB).

Endometrial tissue office biopsies were collected under local anesthesia using an endometrial suction curette (Pipet Curet, CooperSurgical) in the morning after an overnight fast on days 6–8 of a spontaneous period or after an induced withdrawal bleeding to assure that there was no variation caused by the menstrual cycle. One part of each biopsy was snap frozen in liquid nitrogen and stored at −80 °C and one small piece was fixed in 4% paraformaldehyde for histology. After screening and baseline measures, women with PCOS were randomly assigned to one of three groups using our online electronic case report form: (1) lifestyle management alone; (2) metformin + lifestyle; or (3) electroacupuncture + lifestyle, for 16 weeks. In short, all participants received information on lifestyle management. This included an initial counseling session in which they were informed about the importance of weight management, healthy eating and physical activity. All participants received a book with lifestyle advice and received a weekly text message reporting their steps over the previous week and whether they had menstrual bleeding. The metformin group received 500 mg orally, 3× a day for 16 weeks. The dose was increased from 500 mg d^−1^ in week 1 to 1,000 mg d^−1^ in week 2 to the full dose in week 3. Blinding or masking of the intervention was not possible because of the nature of the intervention. Importantly, the assessors were blinded to the patient’s group assignment. For details of interventions, see the published study protocol^60^. In brief, all women received lifestyle management instructions, oral metformin 500 mg 3× daily, in total 1,500 mg d^−1^. Treatment started after baseline measurements, which were repeated after 16 weeks of treatment and snRNA-seq and spatial Stereo-seq were carried out on endometrial tissue biopsies successfully collected at baseline and after 16 weeks of intervention in (1) the lifestyle management alone and (2) the metformin + lifestyle groups.

### Staining and imaging

The fixed endometrial tissue was used to perform immunofluorescence staining of estrogen receptor-α (ER-α). After fixation, the tissues underwent dehydration and were thereafter embedded in paraffin. The paraffin-embedded endometrium was sectioned in 8-mm-thick slices, which were treated with sodium citrate buffer, pH 6.0, for antigen retrieval. After blocking by 5% bovine serum albumin in phosphate-buffered saline (PBS), the sections were incubated with ER-α (rabbit monoclonal, Abcam EPR4097, cat. no. ab108398, lot GR3371387-3, stock 1.009 mg ml^−1^) and EPCAM (mouse monoclonal, Cell Signaling Technology, cat. no. VU-1D9, lot 9) primary antibodies overnight at 4 °C. Then they were incubated with Alexa Fluor-488 (green, Invitrogen, donkey anti-rabbit, cat. no. A21206) and -594 (red, Invitrogen, donkey anti-mouse, cat. no. A21203) conjugated secondary antibodies at 37 °C for 1 h. The sections were then stained with DAPI (Thermo Fisher Scientific, cat. no. 62248) for 5 min. The fluorescent images were taken with an LSM 900 confocal microscope ×63 water (Zeiss). The isotype control images for ER-α were done in the same manner but with the rabbit immunoglobulin G isotype control antibody (Abcam EPR25A, cat. no. ab172730, 1.649 mg ml^−1^) instead of the primary ER-α antibody.

Hematoxylin and eosin (H&E) staining was performed on cryosections made in preparation for spatial transcriptomics. We used the section right before the one used for spatial transcriptomics for all samples besides 031 Baseline, where we used the section that is two after the one used for spatial. Briefly, cryosections were cleared with xylene and rehydrated with ethanol and water, to then be stained with hematoxylin (Dako, cat. no. S3309) for 15 s, washed with tap water until clear and stained with eosin (1:6 dilution, Sigma-Aldrich, cat. no. HT110216) for 10 s. The sections were then submerged in reversed order back to xylene and mounted with Pertex Mounting Media (Histolab Products AB) and covered with a coverslip. The sections were left to dry for 24 h before imaging with an Olympus IX73 widefield inverted microscope by stitching together brightfield images from ×10 magnification.

### Nuclei extraction of frozen endometrial biopsies

Nuclei isolation of snap-frozen endometrial biopsies was modified according to the published tissue in salt tween (TST) method^61^. On ice, a piece of sample (4 × 4 mm^2^) was placed on a precooled Petri dish and chopped with a knife into smaller pieces. The tissue pieces were transferred into a douncer with 1 ml of TST buffer and homogenized 5× with a loose pestle, followed by 8–10× with a tight pestle. The homogenized solution was filtered through a 100-µm filter into a 2-ml tube, followed by filtering of the homogenized solution through a 20-µm filter into a 15-ml tube. An additional 1 ml of TST buffer was used to wash the douncer and the 100-µm and 20-µm filters. An additional 3 ml of 1× ST buffer was added to reach a volume of 5 ml and followed by centrifugation at 4 °C, 300*g* for 4 min. The 4 ml of supernatant was carefully filtered through a 20-µm filter to a new 15-ml tube, followed by centrifugation at 4 °C, 500*g* for 5 min. Thereafter the supernatant was carefully discarded and the pellet was resuspended in 50 µl of nonspecific binding (NSB) buffer and filtered through a 20-µm filter. An additional 50 µl of NSB buffer was added to wash the tube and the filter.

### SnRNA-seq library preparation and sequencing

Following the manufacturer’s protocol, snRNA-seq libraries from isolated nuclei were generated utilizing the Chromium Next GEM Single Cell 3′ Gel Bead Kit (Dual Index) v.3.1 (10x Genomics) and Chromium Next GEM Chip G Single Cell with the goal of capturing 10,000 nuclei per reaction. Library qualities were verified using a 2100 Bioanalyzer (Agilent 4200 TapeStation). Sequencing was performed on the Illumina NovaSeq6000 system at Novogene, with the objective of achieving an average coverage of 25,000 raw reads per nucleus (Supplementary Table 1c). As a result of the inherent challenge in capturing neutrophils using the office biopsies and 10x Genomics platform, the cell type was not captured.

### Tissue preparation for spatial transcriptomics with Stereo-seq

A tissue block with an edge length of <1 cm was rinsed with cold PBS and then embedded in precooled OCT (Sakura) using dry ice for setting of OCT. Three to four serial cryosections of 10-µm thickness were cut from the OCT-embedded samples for H&E staining and Stereo-seq library preparation. Brightfield images of the H&E samples were acquired using a Motic microscope scanner for histopathological evaluation.

### Quality control of RNA from OCT-embedded samples

From each OCT-embedded sample, 100- to 200-µm-thick sections were cut for extraction of total RNA using the RNeasy Mini Kit (QIAGEN) according to the manufacturer’s protocol. The RNA integrity number (RIN) was determined using the Bioanalyzer (Agilent 4200 TapeStation). Samples that qualified for the transcriptomic study had a RIN between 7.3 and 8.0.

### Stereo-seq library preparation and sequencing

The spatial transcriptomic RNA library was prepared using Stereo-seq chips (BGI Research) with an area of 1 cm^2^. The capture spots had a diameter of 220 nm and a center-to-center distance of 500 nm. Each Stereo-seq capture probe contained a 25-bp coordinate identity barcode, a 10-bp molecular identity barcode and a 22-bp poly(T) tail for in situ messenger RNA capture. A 10-µm cryosection from the OCT-embedded tissue was quickly placed on the chip, incubated at 37 °C for 5 min and then fixed in precooled methanol at −20 °C for 30 min. The fixed tissue sections were stained with Qubit single-stranded DNA dye (Thermo Fisher Scientific) to obtain the nuclei location information. Tissue sections were permeabilized for 6 min at 37 °C in 100 μl of 0.1% pepsin in 0.01 mol l^−1^ of HCl buffer, followed by a wash with 0.1× saline-sodium citrate supplemented with 0.05 U μl^−1^ of RNase inhibitor. The RNA released from the permeabilized tissue was captured in situ by capture oligo-probes and was reverse transcribed at 42 °C for 3 h. The tissue sections were then digested with a tissue removal buffer at 55 °C for 10 min. The complementary DNA-containing chip was incubated overnight at 55 °C in 400 μl of cDNA release buffer. The released cDNA was further purified and amplified using cDNA amplification primer. Approximately 20 ng of cDNA was fragmented, amplified and purified to generate each sequencing library. Sequencing was performed using the pair-end 50 + 100 bp strategy on an MGI DNBSEQ-Tx sequencer.

### Endometrium snRNA-seq analysis

The raw sequencing reads from the FASTQ files were aligned to a reference transcriptome (GRCh38-2020-A) including introns using Cell Ranger Software (v.6.1.1). Filtered reads from Cell Ranger were transformed into Seurat object for initial quality control with Seurat (v.4) and processed individually to remove low-quality nuclei. Nuclei with <500 genes, >10% mitochondrial reads, >5% ribosomal reads and >1% hemoglobin reads were filtered out and genes expressed in <3 nuclei were removed. Duplicates and multiples were removed with scDblFinder (v.1.18). The filtered Seurat objects were integrated using the sctransform method v.2.0 from the Satija lab^62^. Seurat’s SelectIntegrationFeatures with 2,500 features and FindIntegrationAnchors with the default settings were used to identify anchors to integrate the samples. Integration was performed using IntegrateData and sctransform as the normalization method and used for clustering with uniform manifold approximation and projection (UMAP). After clustering with sctransform, the count matrix was normalized and transformed using Seurat log(normalize).

#### Cell-type annotation and subclustering

Cell-type annotation was performed manually by comparing marker gene expression with previous published work and applying FindAllMarkers to sctransform clusters to examine the most highly expressed genes in each cluster. The seven main cell-type clusters epithelial, stromal, SMCs, immune including lymphoid and myeloid cells, endothelial and lymphatic cells were further subdivided from the main Seurat object and reintegrated using the sctransform method as described above. Epithelial, stromal and endothelial cells were manually annotated, whereas immune cells were manually annotated and validated using SingleR (v.2.6) and the Monaco immune data as a reference dataset. Sctransform was applied to subcluster PCOS induced and noninduced per main cell type.

#### Differential gene expression analysis and GO enrichment analysis of snRNA-seq

DEGs were identified pairwise by applying FindMarkers and the MAST method^63^ (v.1.3) at baseline comparing the control group with the PCOS group, and the effect of intervention by comparing the PCOS-baseline with the PCOS-metformin group and the PCOS-lifestyle group, respectively. The identified DEGs were categorized into two groups based on whether gene expression was up- or downregulated and on a positive or negative log_2_(fold-change) (log_2_(FC)) before importing them into the clusterProfiler package (v.4.12.2) to perform a functional enrichment analysis of the biological process ontology with enrichR (v.3.2) using the compareCluster function. This generated two sets of GO output tables with either up- or downregulated GOs per comparison and cellular subpopulation. The GO tables from the different comparisons and cell types were merged and manually curated to identify GO terms relevant to PCOS and endometrium and to identify GO terms that reverse after treatment.

### CellChat: snRNA-seq cell–cell communication analysis

Cell–cell communication analysis was performed using CellChat (v.2.1.2)^64^ with NMF (v.0.27) and reticulate (v.1.38). All identified subclusters of the epithelium, stroma, immune system and endothelium or lymphatic system were projected on to the original Seurat object of all cell nuclei. CellChat objects with the group labels control and PCOS-baseline, as well as metformin and lifestyle treatment, were generated from the Seurat object and the log(normalized) RNA matrix. In this way, four CellChat objects were generated, which were processed individually before being merged into three groups for comparisons: (1) control versus PCOS at baseline; (2) PCOS: baseline versus 16 weeks of metformin; and (3) PCOS: baseline versus 16 weeks of lifestyle. Up- and downregulated genes were identified using default settings (percentage expression >25%, log(FC) > 0.5, adjusted *P* < 0.05) before prediction of ligand–receptor interactions using CellChatDB human. Communication probabilities were calculated using computeCommunProb with population.size to account for cell clusters of different sizes, followed by the computeCommunPropPathway to predict signaling pathways and the relative strength of these pathways based on predicted strength. Based on predicted ligand and receptor expression, information flow was mapped and labeled as either outgoing signaling indicative of ligand expression or incoming signaling indicative of receptor expression. Taken together, CellChat enabled the prediction of which cell subpopulations interact based on the ligand and receptor expression predicted from the gene expression data and to which signaling pathways the interactions belong. The individual CellChat objects were merged to facilitate comparison of the communication probabilities of cell-type interactions between the groups. In this way, it was possible to investigate how the relative strength of ligand–receptor interactions differed between cell types in the groups. The results obtained showed both shared pathways between the groups and pathways that were not shared, suggesting that pathways are up- or downregulated in PCOS and whether treatment affects them. Genes encoding the ligand–receptor pairs with predicted interaction were extracted and used to visualize the interaction. When comparing the relative strength of interactions between cases and controls, only ligand–receptor pairs and pathways with identified DEGs were considered by MAST-based DEG analysis. DEGs were further filtered by comparing them with DEGs found in PCOS versus metformin and PCOS versus lifestyle to discover ligand and receptor treatment-responsive genes.

### Endometrium Stereo-seq analysis

Stereo-seq Analysis Workflow (SAW) (v.5.5.3) and anndata (v.0.7.5.6) were utilized to process the raw data generated from Stereo-seq, including the FASTQ and mask file storing the coordinate ID of the chips^65^. Stereopy (v.1.1.0) was used to bin the data to bin30 and generated gene expression matrices were converted to Seurat objects for further downstream processing and harmonization with the existent snRNA-seq dataset. The data were filtered to remove bins with >20% mitochondrial reads, >5% ribosomal reads, >1% hemoglobin reads, genes expressed in <3 bins and bins expressing >250 or <10,000 mapped reads. The data were normalized using both sctransform and log(normalization), of which sctransform was used only for clustering the data. The clusters were annotated using the snRNA-seq dataset with the major cell-type annotation as a reference, of which the spatial dataset was projected on to using the Seurat unimodal UMAP projection MapQuery. The annotated spatial object was filtered to include only bins with a prediction score >0.5. The annotation of the spatial object was used as a guide when identifying regions of the spatial object, including both epithelial and stroma cell types on which to project gene marker expression.

### Candidate etiological cell types via CELLEX and CELLECT

GWAS summary statistics for nine given traits (Supplementary Table 5a) and cell-type expression specificity estimates derived from our snRNA-seq data are the input data for CELLECT (v.1.3)^51^. The output is a list of candidate etiological cell types for a given trait. CELLEX (v.1.2.2) calculates robust estimates of expression specificity and was run separately on the control and PCOS snRNA-seq gene expression matrices and on the combined (PCOS + control) matrices to calculate gene expression specificities for each subcell type. The cell-type specificity matrix was used together with GWASs as input for CELLECT–MAGMA covariate analyses using default parameters.

### Statistical analyses

Statistical analyses of the clinical variables were performed in IBM SPSS Statistics v.29.0.2.0 or R v.4.4.0. To examine differences in nuclei proportion, first the Kolmogorov–Smirnov and Shapiro–Wilk tests were used to check the data distribution. As most of the data were not normally distributed, the nonparametric, two-sided Mann–Whitney *U*-test was used for comparisons between women with and women without PCOS at baseline and the two-sided Wilcoxon’s signed-rank test was used to investigate changes from baseline to after 16 weeks of intervention (metformin and lifestyle, respectively) in women with PCOS. To correct for multiple testing, Benjamini–Hochberg (BH)-adjusted *P* < 0.05 was used to determine whether the difference was significant. For DEG analyses, an adjusted *P* < 0.05, using BH, with a log_2_(FC) > 0.5 or <−0.5 after two-sided MAST testing and minimal fraction of gene expression in either population, >0.25 was considered significant. For GO enrichment analysis, the most enriched biological processes with at least three genes and an adjusted *P* < 0.05, that is, false discovery rate (FDR), after one-sided over-representation analysis were considered significant and reported. In the CELLECT and CELLEX analysis, significant cell types were identified using by trait Bonferroni’s *P*-value threshold of *P* < 0.05. Spearman’s rank correlation was used to assess the relationship between the averaged gene expression of DEGs per cell type and sample and clinical variables that differed between women with and women without PCOS. Relationships with *R*_S_ > 0.5 or *R*_S_ < −0.5 and *P* < 0.05 were considered significant. For the snRNA-seq, targeting ~10,000 nuclei per individual, with ~25,000 raw reads per nucleus and our sample size, we expected to detect differences with a power of ~80–90% and a high effect size with a mean of 0.7–0.9 and an s.d. of 0.2.

### Reporting summary

Further information on research design is available in the Nature Portfolio Reporting Summary linked to this article.

## Online content

Any methods, additional references, Nature Portfolio reporting summaries, source data, extended data, supplementary information, acknowledgements, peer review information; details of author contributions and competing interests; and statements of data and code availability are available at 10.1038/s41591-025-03592-z.

## Supplementary information

Supplementary Information Supplementary Tables 1–6.

Reporting Summary

Supplementary Table 1–6 Table 1**a**, Clinical characteristics of women with and women without PCOS at baseline and change from baseline to after 16 weeks of intervention in PCOS. Difference between PCOS versus controls. Statistical analyses were performed by Mann–Whitney *U*-test (#) and Wilcoxon’s signed-rank test (##) for changes from baseline to after 16 weeks of metformin and lifestyle treatment, respectively. **b**, Anonymized individual clinical information. **c**, Details of number of reads per nuclei per sample. **d**, Number of nuclei per individual in each subpopulation. **e**, Proportions of nuclei per sample in each subpopulation and proportion of nuclei in each group. **f**, Proportion of nuclei in PCOS-endometrium with and without Provera-induced bleeding. **g**, Comparison of nuclei proportion in the present dataset and in the HECA study (1). **h,** Details of quality control of Stereo-seq data in each sample. Table 2**a**, DEGs between women with PCOS and controls. **b**,**c**, DEG changes from baseline to after 16 weeks of metformin treatment (**b**) or lifestyle management (**c**) in women with PCOS. Statistics for DEG analyses using BH and an adjusted *P* < 0.05 was considered significant. Table 3**a**, Enriched GO for biological processes (BPs) based on DEGs comparing women with PCOS with controls at baseline. **b**,**c**, Enriched GO of BPs on DEGs based on changes from baseline to 16 weeks of metformin treatment (**b**) or lifestyle management (**c**) in women with PCOS. Table 4 a, CellChat controls. **b**, PCOS. **c**, Metformin. **d,** Lifestyle management. Table 5**a**, List of sources for GWAS datasets used in the CELLEX. **b**, CELLECT output for subclusters in PCOS and controls. **c**, CELLECT output for subclusters in PCOS. **d**, CELLECT output for subclusters in controls. Table 6 Spearman’s correlation analyses between the calculated averaged gene expression of DEGs per cell type and sample and clinical variables that differ between women with PCOS and controls. Relationships with *R*_S_ > 0.5 or *R*_S_ < −0.5 and *P* < 0.05 were considered significant.

## Source data

Source Data Fig. 2 Nuclei frequencies per group in epithelium cluster.

Source Data Fig. 3 Nuclei frequencies per group in stroma cluster.

Source Data Fig. 4 Nuclei frequencies per group in immune and endothelial or lymphatic cluster.

Source Data Extended Data Fig. 2 Nuclei frequencies of spatial transcriptomic per participant in main cell types.

## Data Availability

Individual raw sequencing reads of snRNA-seq data are available in the European Genome–Phenome Archive, accession no. EGAD50000001017. Processed sequencing data and anonymized human data will be provided by the corresponding author E.S.-V. upon request. Source data are provided with this paper.

## Code availability

All code developed and used for data analysis in the present study is available at https://github.com/ReproductiveEndocrinologyMetabolism/Endo.R.
