# Supplementary material for: Single-cell profiling of the human endometrium in polycystic ovary syndrome
Source: Nat Med. 2025 Mar 20;31(6):1925–38. doi: 10.1038/s41591-025-03592-z (PMC12176659; doi:10.1038/s41591-025-03592-z)
Supplement: Supplementary file 1 — Supplementary Tables 1–6. [file 41591_2025_3592_MOESM1_ESM.pdf]

nature medicine

Supplementary information https://doi.org/10.1038/s41591-025-03592-z

Single-cell profiling of the human endometrium in polycystic ovary syndrome

In the format provided by the authors and unedited

Legends - Supplementary Tables

Table 1a. Clinical characteristics of women with and without PCOS at baseline and change from baseline to after 16 weeks of intervention in PCOS. Difference between PCOS versus controls. Statistical analyses were performed by Mann Whitney U test (#); and Wilcoxon signed rank test (##) for changes from baseline to after 16 weeks of metformin and lifestyle treatment, respectively. **b.** Anonymized individual clinical information. **c.** Details of number of reads per nuclei per sample. **d.** Number of nuclei per individual in each subpopulation. **e.** Proportions of nuclei per sample in each subpopulation and proportion of nuclei in each group. **f.** Proportion of nuclei in PCOS-endometrium with and without Provera-induced bleeding. **g.** Comparison of nuclei proportion in the present data set and in the HECA study (1). **h.** Details of quality control of Stereo-seq data in each sample.

Table 2a. Differentially expressed genes (DEGs) between women with PCOS and controls. **b.** DEG changes from baseline to after 16 weeks of metformin treatment or **c.** lifestyle management in women with PCOS. Statistics for DEG analyses using Benjamin-Hochberg and an adjusted p-value <0.05 was considered significant.

Table 3a. Enriched gene ontology (GO) for biological processes (BP) based on DEGs comparing women with PCOS with controls at baseline. **b.** Enriched GO of BPs on DEGs based on changes from baseline to 16 weeks of metformin treatment or **c.** lifestyle management in women with PCOS.

Table 4a. CellChat controls, **b.** PCOS, **c.** metformin and **d.** lifestyle management.

Table 5a. List of sources for GWAS datasets used in the CELLEX. **b.** CELLECT output for subcluster in PCOS and controls. **c.** CELLECT output for subcluster in PCOS. **d.** CELLECT output for subcluster in controls.

Table 6. Spearman's correlation analyses between the calculated averaged gene expression of DEGs per cell type and sample and clinical variables that differ between women with PCOS and controls. Relationships with R_S>0.5 or R_S<-0.5 and a p-value < 0.05 were considered significant.

Reference

1. Marečková M, Garcia-Alonso L, Moullet M, Lorenzi V, Petryszak R, Sancho-Serra C, et al. An integrated single-cell reference atlas of the human endometrium. Nat Genet. 2024.
