# Supplementary material for: Single-cell profiling of the human endometrium in polycystic ovary syndrome
Source: Nat Med. 2025 Mar 20;31(6):1925–38. doi: 10.1038/s41591-025-03592-z (PMC12176659; doi:10.1038/s41591-025-03592-z)
Supplement: Supplementary file 2 — Reporting Summary [file 41591_2025_3592_MOESM2_ESM.pdf]

nature portfolio

Corresponding author(s): Elisabet Stener-Victorin

Last updated by author(s): Feb 2, 2025

# Reporting Summary

Nature Portfolio wishes to improve the reproducibility of the work that we publish. This form provides structure for consistency and transparency in reporting. For further information on Nature Portfolio policies, see our Editorial Policies and the Editorial Policy Checklist.

## Statistics

For all statistical analyses, confirm that the following items are present in the figure legend, table legend, main text, or Methods section.

| n/a | Confirmed | |
|---|---|---|
| ☐ | ☒ | The exact sample size (*n*) for each experimental group/condition, given as a discrete number and unit of measurement |
| ☐ | ☒ | A statement on whether measurements were taken from distinct samples or whether the same sample was measured repeatedly |
| ☐ | ☒ | The statistical test(s) used AND whether they are one- or two-sided<br>*Only common tests should be described solely by name; describe more complex techniques in the Methods section.* |
| ☐ | ☒ | A description of all covariates tested |
| ☐ | ☒ | A description of any assumptions or corrections, such as tests of normality and adjustment for multiple comparisons |
| ☐ | ☒ | A full description of the statistical parameters including central tendency (e.g. means) or other basic estimates (e.g. regression coefficient) AND variation (e.g. standard deviation) or associated estimates of uncertainty (e.g. confidence intervals) |
| ☐ | ☒ | For null hypothesis testing, the test statistic (e.g. $F$, $t$, $r$) with confidence intervals, effect sizes, degrees of freedom and $P$ value noted<br>*Give P values as exact values whenever suitable.* |
| ☐ | ☒ | For Bayesian analysis, information on the choice of priors and Markov chain Monte Carlo settings |
| ☐ | ☒ | For hierarchical and complex designs, identification of the appropriate level for tests and full reporting of outcomes |
| ☒ | ☐ | Estimates of effect sizes (e.g. Cohen's *d*, Pearson's *r*), indicating how they were calculated |

*Our web collection on statistics for biologists contains articles on many of the points above.*

## Software and code

Policy information about availability of computer code

| | |
|---|---|
| Data collection | No software was used for data collection. |
| Data analysis | Data Analysis:<br>Alignment and quantification of snRNA-seq data:<br>Cell Ranger Software v.6.1.1<br>Alignment and quantification of spatial RNA-seq:<br>SAW v5.5.3; Stereopy v.l.1.0<br>Downstream analysis of snRNA-seq and spatial RNA-seq:<br>Seurat v.4; scDblFinder v.l.18; sctransform v.2.0; SingleR v.2.6; MAST v.1.3; clusterProfiler v.4.12.2; enrichR v.3.2; CellChat v.2.1.2; NMF v.0.27; CELLEX v.l.2.2; CELLECT v.1.3., anndata (v. 0.7.5.6)<br>Costume code available at GitHub upon publication: https://github.com/ReproductiveEndocrinologyMetabolism/ENDO.R |

For manuscripts utilizing custom algorithms or software that are central to the research but not yet described in published literature, software must be made available to editors and reviewers. We strongly encourage code deposition in a community repository (e.g. GitHub). See the Nature Portfolio guidelines for submitting code & software for further information.

## Data

Policy information about availability of data

All manuscripts must include a data availability statement. This statement should provide the following information, where applicable:

- Accession codes, unique identifiers, or web links for publicly available datasets
- A description of any restrictions on data availability
- For clinical datasets or third party data, please ensure that the statement adheres to our policy

The raw sequencing reads from the FASTQ files were aligned to a reference transcriptome GRCh38-2020-A.
Individual raw sequencing reads of snRNA-seq data are available in The European Genome-Phenome Archive (EGA) accession number EGAD50000001017. Processed sequencing data and anonymized human data will be provided by the corresponding author Elisabet Stener-Victorin upon request.

## Research involving human participants, their data, or biological material

Policy information about studies with human participants or human data. See also policy information about sex, gender (identity/presentation), and sexual orientation and race, ethnicity and racism.

| | |
|---|---|
| Reporting on sex and gender | Information about sex and gender is not specifically reported. All included participants are referred to as women. |
| Reporting on race, ethnicity, or other socially relevant groupings | All participants were of European origin and this is reported in Supplementary Table 1b. No other grouping is reported. |
| Population characteristics | 12 women diagnosed with polycystic ovary syndrome (PCOS) and 5 women of similar age, weight and BMI referred to as non-PCOS, women without PCOS or controls.<br>Clinical characteristics on group level is reported in Supplementary Table 1a and on individual level in Supplementary Table 1b. |
| Recruitment | The women were recruited via online advertisements and examined at Women's Health Research Unit at the Karolinska University Hospital, Stockholm. Prior to any assessments, women received oral and written information and provided written informed consent. All women included in the study were ≥ 18to ≤ 40 years of old with a BMI ≥ 25. PCOS diagnosis was set according to the revised 2003 Rotterdam criteria, excluding any endocrine related disorder58. Transvaginal ultrasound measured number of antral follicles (2 – 9 mm), ovarian volume (> 10 cm3), and endometrial thickness (mm). Women with PCOS had irregular menstrual cycles >35 days. Ferriman-Gallwey score was used to assess hirsutism with a score >4 considered as clinical hyperandrogenism. The control subjects did not differ from PCOS subjects in terms of age, weight and BMI. In addition, controls had less than 12 antral follicles (2 – 9 mm), an ovarian volume of less than 10 cm3, regular menstrual cycles (+/- 28 days) and a Ferriman-Gallwey score ≤ 4. Both women with and without PCOS had no other gynecological diseases (e.g. uterine fibroids, endometriosis), were neither pregnant nor breastfeeding in the last 6 months and were not taking any medication or hormonal treatment, including oral contraceptives, for at least 3 months before the baseline examinations, except for some women who took Provera 10 mg for 7 days to induce bleeding.<br>Self-selection bias: Only women with who donated endometrial tissue biopsies were included. As this was random we do not expect that it affect the outcome. |
| Ethics oversight | The study adheres to good clinical practice and follows the Declaration of Helsinki. Approval has been granted by the Regional Ethical Review Board of Stockholm, Sweden Dnr: 2015/1656-31/2 with amendment Dnr: 2024-00633-02 and is approved by the Medical Products Agency: EUCT: 2024-514505-64-00 and registered at Clinicaltrials.gov: NCT02647827. Prior to any assessments, women received oral and written information and provided written informed consent. |

Note that full information on the approval of the study protocol must also be provided in the manuscript.

# Field-specific reporting

Please select the one below that is the best fit for your research. If you are not sure, read the appropriate sections before making your selection.

☒ Life sciences ☐ Behavioural & social sciences ☐ Ecological, evolutionary & environmental sciences

For a reference copy of the document with all sections, see nature.com/documents/nr-reporting-summary-flat.pdf

# Life sciences study design

All studies must disclose on these points even when the disclosure is negative.

| | |
|---|---|
| Sample size | No statistical method were used to predetermine sample size.<br>In total 27 endometrial biopsies from 17 donors (controls, n = 5; PCOS baseline n=12, after 16 weeks of intervention n=l0).<br>Estimates were made based on our previous experience and sample availability.<br>With our sample size we expect to detect differences with a power of ~80-90% and a high effect size with a mean of o. 7-0.9 and a standard deviation of 0.2. |

Data exclusions | No data were excluded from analysis.

Replication | The main snRNA-seq findings has succesfully been technically and biologically replicated by spatial transcriptomic using Stero-seq in two PCOS, two controls and in one metformin and one lifestyle management sample.

Randomization | After screening and baseline measures, women with PCOS were randomly assigned to one of three groups using our online electronic case report form: 1) Lifestyle management alone; 2) Metformin + lifestyle or 3) Electroacupuncture + lifestyle, for 16 weeks. In short, all participants received information on lifestyle management. This included an initial counseling session in which they were informed about the importance of weight management, healthy eating and physical activity. All participants received a book with lifestyle advice and received a weekly text message reporting their steps over the previous week and whether they had menstrual bleeding. The metformin group received 500 mg orally, three times a day for 16 weeks. The dose was increased from 500 mg/day in week 1 to 1000 mg/day in week 2 to the full dose in week 3. Blinding or masking of the intervention was not possible because of the nature of intervention. Importantly, the assessors were blinded to the patient's group assignment. For details of interventions, see published study protocol (PMID: 30612112). In brief, all women received lifestyle management instructions, oral metformin 500 mg three times daily, in total 1500 mg per day. Treatment started after baseline measurements which was repeated after 16 weeks of treatment and snRNA-sequencing and spatial STEREO-seq was done on endometrial tissue biopsies successfully collected at baseline and after 16 weeks of intervention in the 1) Lifestyle management alone and 2) Metformin + lifestyle groups.

Blinding | Blinding or masking of the intervention was not possible because of the nature of the interventions. Importantly, the assessors were blinded to the patient's group assignment.

# Reporting for specific materials, systems and methods

We require information from authors about some types of materials, experimental systems and methods used in many studies. Here, indicate whether each material, system or method listed is relevant to your study. If you are not sure if a list item applies to your research, read the appropriate section before selecting a response.

## Materials & experimental systems

| n/a | Involved in the study |
|---|---|
| ☐ | ☒ Antibodies |
| ☒ | ☐ Eukaryotic cell lines |
| ☒ | ☐ Palaeontology and archaeology |
| ☒ | ☐ Animals and other organisms |
| ☐ | ☒ Clinical data |
| ☒ | ☐ Dual use research of concern |
| ☒ | ☐ Plants |

## Methods

| n/a | Involved in the study |
|---|---|
| ☒ | ☐ ChIP-seq |
| ☒ | ☐ Flow cytometry |
| ☒ | ☐ MRI-based neuroimaging |

## Antibodies

Antibodies used | ER-alpha (Rabbit Monoclonal, Abcam EPR4097, ab108398, LOT: GR3371387-3, stock 1.009 mg/mL) and EPCAM (Mouse Monoclonal, Cell Signaling Technology, VU-1D9, LOT: 9) primary antibodies overnight at 4C°. Then incubated with Alexa Fluor 488 (green, Invitrogen donkey anti-rabbit A21206) and 594 (red, Invitrogen donkey anti-mouse A21203)-conjugated secondary antibodies at 37C° for 1hr. The sections were then stained with 4',6-diamidino-2-phenylindole (DAPI) (Thermo Scientific 62248) for 5 min. The fluorescent images were taken with LSM 900 confocal microscope 63x water (Zeiss).
Hematoxylin and eosin (H&E) staining was performed on cryo-sections made in preparation for spatial transcriptomics. Cryosections were cleared with xylene and re-hydrated with ethanol and water to then be stained with hematoxylin (Code S3309, Dako) for 15 seconds, washed with tap water until clear, and stained with eosin (1:6 dilution, HT110216, Sigma-Aldrich) for 10 seconds.

Validation | The isotype control images for ER-alpha were done in the same manner but with the Rabbit IgG Isotype Control antibody (Abcam EPR25A, ab172730, stock 1.649 mg/mL) instead of the primary ER-alpha antibody.

## Clinical data

Policy information about clinical studies
All manuscripts should comply with the ICMJE guidelines for publication of clinical research and a completed CONSORT checklist must be included with all submissions.

Clinical trial registration | Clinicaltrials.gov: NCT02647827 and Medical Products Agency: EUCT: 2024-514505-64-00.

Study protocol | Published study protocol (PMID: 30612112).

Data collection | Baseline examination before start of treatment: Participants arrives fasted in the morning to the Women's Health Unit at Karolinska University Hospital in day 6-8 of the menstrual cycle. A doctor perform a gynecological examination, including an ultrasound of the ovary and uterus, and also sampling from endometrium, a so-called endometrium biopsy, which will be done during local anesthetics with a fine instrument. Then a blood sample (20ml) is taken for analysis of hormone levels.

| | |
|---|---|
| Outcomes | The purpose of this study is twofold. First, we aim to gain deeper insight into the pathophysiology of PCOS through a cross-sectional study comparing women with PCOS to women without PCOS, with similar age, weight, and BMI, across primary and secondary outcome variables. Second, we will conduct a randomized controlled trial in women with PCOS to compare the effectiveness of lifestyle management alone versus lifestyle management combined with acupuncture or metformin treatment on whole-body glucose homeostasis and sex steroid levels. Moreover, we perform single-nucleus RNA sequencing and spatial transcriptomics to create a cellular and transcriptional atlas of the proliferative-phase endometrium in women with and without PCOS, and to define changes in PCOS-specific endometrial signatures after 16 weeks of metformin and lifestyle management. |

## Plants

| | |
|---|---|
| Seed stocks | N/A |
| Novel plant genotypes | N/A |
| Authentication | N/A |
